# Overlooked Vital Role of Persistent Algae‐Bacteria Interaction in Ocean Recalcitrant Carbon Sequestration and Its Response to Ocean Warming

**DOI:** 10.1111/gcb.17570

**Published:** 2024-11-27

**Authors:** Hanshuang Zhao, Zenghu Zhang, Shailesh Nair, Hongmei Li, Chen He, Quan Shi, Qiang Zheng, Ruanhong Cai, Genming Luo, Shucheng Xie, Nianzhi Jiao, Yongyu Zhang

**Affiliations:** ^1^ Qingdao New Energy Shandong Laboratory, Key Laboratory of Biofuels, Shandong Provincial Key Laboratory of Energy Genetics Qingdao Institute of Bioenergy and Bioprocess Technology, Chinese Academy of Sciences Qingdao China; ^2^ University of Chinese Academy of Sciences Beijing China; ^3^ Shandong Energy Institute Qingdao China; ^4^ State Key Laboratory of Heavy Oil Processing China University of Petroleum Beijing China; ^5^ State Key Laboratory of Marine Environmental Science Xiamen University Xiamen China; ^6^ State Key Laboratory of Biogeology and Environmental Geology, School of Earth Sciences China University of Geosciences Wuhan China

**Keywords:** carbon sequestration, FT‐ICR MS, metagenomics, ocean warming, recalcitrant dissolved organic carbon, Synechococcus‐bacteria interactions

## Abstract

Long‐term carbon sequestration by the ocean's recalcitrant dissolved organic carbon (RDOC) pool regulates global climate. Algae and bacteria interactively underpin RDOC formation. However, on the long‐term scales, the influence of their persistent interactions close to in situ state on ocean RDOC dynamics and accumulation remains unclear, limiting our understanding of the oceanic RDOC pool formation and future trends under global change. We show that a *Synechococcus*‐bacteria interaction model system viable over 720 days gradually accumulated high DOC concentrations up to 84 mg L^−1^. Concurrently, the DOC inertness increased with the RDOC ratio reaching > 50%. The identified *Synechococcus*‐bacteria‐driven RDOC molecules shared similarity with over half of those from pelagic ocean DOC. Importantly, we provide direct genetic and metabolite evidence that alongside the continuous transformation of algal carbon by bacteria to generate RDOC, *Synechococcus* itself also directly synthesized and released RDOC molecules, representing a neglected RDOC source with ~0.2–1 Gt y^−1^ in the ocean. However, we found that although ocean warming (+4°C) can promote algal and bacterial growth and DOC release, it destabilizes and reduces RDOC reservoirs, jeopardizing the ocean's carbon sequestration capacity. This study unveils the previously underestimated yet significant role of algae and long‐term algae‐bacteria interactions in ocean carbon sequestration and its vulnerability to ocean warming.

## Graphical abstract

We demonstrate that long‐term algae‐bacteria interactions contribute significantly to the marine recalcitrant dissolved organic carbon (RDOC) pool, which is essential for ocean carbon sequestration and climate change mitigation. We also provide the first direct evidence that algae can directly synthesize and release RDOC. However, global warming may weaken this important carbon sequestration process by destabilizing RDOC reservoirs. These findings highlight the underestimated yet significant role of algae and long‐term algae‐bacteria interactions in ocean carbon sequestration and its vulnerability to future ocean warming.

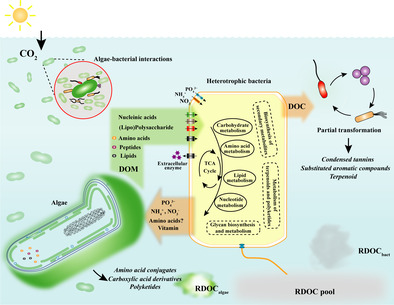

## 1 Introduction

Marine dissolved organic carbon (DOC) represents one of the largest actively cycling carbon reservoirs on Earth, nearly equivalent in size to the atmospheric CO_2_ pool (Jiao et al. 2010; Zhang et al. 2018). 60%–95% fraction of this DOC pool consists of recalcitrant DOC (RDOC), which is sequestered over millennia in the ocean and plays a pivotal role in climate regulation (Hansell 2013; Middelboe and Lundsgaard 2003; Zhang et al. 2018). The continuous production and accumulation of the RDOC are primarily driven by synergistic interactions between algae and heterotrophic bacteria (Nair et al. 2022; Seymour et al. 2017). Algae release a substantial amount of photosynthetically fixed carbon as DOC, estimated around 10%–50% of primary production (Muhlenbruch et al. 2018; Thornton 2014). A major fraction of this freshly released DOC is labile (LDOC), which is rapidly utilized by heterotrophic bacteria and converted into inorganic nutrients (such as CO_2_, nitrate, and phosphate) through remineralization. These nutrients then become available in the water column and can be taken up by algae to support their growth and photosynthetic activity (Christie‐Oleza et al. 2017; Seymour et al. 2017). During utilization, heterotrophic bacteria constantly transform and condense a fraction of the LDOC into complex RDOC structures, through the microbial carbon pump (MCP) framework (Jiao et al. 2014; Zhang et al. 2018). Thereby, the synergistic interaction between algae and heterotrophic bacterial consortia enables a constant influx and sequestration of carbon over longer timescales into the ocean's vast RDOC reservoir.

But how well do we truly understand the contribution of their continuous interaction in driving RDOC formation and accumulation? Our understanding of RDOC formation in the ocean largely hinges on studies focused on the bacterial conversion of carbon sourced from dead algal cells or algal exudates (Wang et al. 2022; Zhao et al. 2019; Zheng et al. 2020). However, long‐term, close‐knit, and ever‐changing interactions between live algae and bacteria are ubiquitous in marine environments (Nair et al. 2022; Seymour et al. 2017; Zhang, Nair et al. 2021). This continuous, dynamic interaction between algae and associated bacteria can synergistically influence each other's physiology and carbon processing (Nair et al. 2022), ultimately affecting the fate and persistence of DOC in the ocean. For instance, the interaction between the diatom *Phaeodactylum tricornutum* and associated bacteria enhanced the secretion of mucin‐like proteinaceous substances by the diatom (Buhmann et al. 2016). Moreover, algae‐bacterial interactions also enhanced the algal production of the carboxyl‐rich alicyclic molecule (CRAM)‐like organic matter (Liu et al. 2021). Despite such sporadic evidence, the dynamics (especially the extent) of RDOC genesis driven specifically by long‐term living algae‐bacteria interactions remain largely unexplored, undermining a comprehensive understanding of the marine carbon cycle and carbon sequestration. Additionally, while the significance of bacteria in reworking algae‐derived LDOC into RDOC is established, the ability of certain algae, including the ubiquitous marine picocyanobacterium *Synechococcus* to produce recalcitrant CRAM and humic‐like substances (Chrost and Faust 1983; Zhang, Tang et al. 2021; Zhao et al. 2017), suggests the potential of algae to release intrinsic RDOC molecules directly. Nevertheless, the potential for mixotrophic algae to consume DOC compounds (Bronk et al. 2007; Wu et al. 2022), suggests an additional pathway for direct algal release of RDOC formation analogous to the MCP. However, the poor differentiation between algal‐ and bacterial‐derived RDOC and the lack of direct molecular and genetic evidence of algal‐derived RDOC in previous studies has inadvertently overlooked or underestimated the potential direct contribution of algae to marine RDOC inventories.

Given the persistent interaction between algae and bacteria and the extremely stable nature of RDOC, theoretically, it is plausible that the concentration of RDOC in the ocean could gradually rise to substantially elevated levels, without accounting for the influence of other organisms (such as protozoa and viruses) and environmental factors. However, many other factors may limit the formation and accumulation of RDOC. For instance, elevated concentrations of certain DOC compounds may incur toxicity and inhibit algal growth and DOC release (Christie‐Oleza et al. 2017). Changes in environmental variables can modulate algal physiology, bacteria community composition, and their subsequent DOC cycling (Seymour et al. 2017). Importantly, changing ocean conditions such as warming have also been predicted to elevate the microbial degradation of labile, semi‐labile and semi‐recalcitrant DOC components (Lønborg et al. 2020). While the persistent synergistic interactions between algae and bacteria significantly influence ocean carbon cycling, to what extent long‐term algae‐bacteria interactions contribute to RDOC dynamics in the absence of the top‐down regulatory pressures (protozoa and viruses), and how their RDOC dynamics respond to changing oceanic conditions is unknown.

Marine picocyanobacterial genera, *Synechococcus* is the most ubiquitous algal group in the ocean, responsible for up to 20% of marine primary production and contributes substantially to oceanic DOC inventory (Flombaum et al. 2013; Liang et al. 2017). Moreover, its ecological importance and role in carbon cycling are projected to grow under anticipated climatic changes (Flombaum et al. 2013). Additionally, studies have observed that over 40% of *Synechococcus* in the in situ ocean are closely associated with bacteria (Malfatti and Azam 2009). Moreover, *Synechococcus* and bacteria share a close metabolic exchange (Raina et al. 2023), and their autotrophic and heterotrophic relationships represent the most fundamental and common microbial associations in the ocean, forming the basis of the marine microbial cycle (Seymour et al. 2017). In our previous study, we successfully established a long‐term algae‐bacteria mutualistic model system between *Synechococcus* strain PCC7002 and a natural heterotrophic bacterial community. Notably, both the algae and bacteria demonstrated long‐term robust growth through reciprocal nutrient generation, without any supplementation of exogenous nutrients (Nair et al. 2022; Zhang, Nair et al. 2021). Leveraging this ideal model, we hypothesize that the synergistic carbon processing in long‐term *Synechococcus*‐heterotrophic bacteria interaction system may promote the continuous accumulation of RDOC to a potentially high concentration. This is due to the continuous release of organic carbon, potentially containing some intrinsic RDOC molecules by the algae and the simultaneous transformation of the algal carbon into more recalcitrant forms by the heterotrophic bacteria. To investigate this, here we evaluated the dynamics of RDOC formation and accumulation in the *Synechococcus*‐heterotrophic bacterial mutualistic model system over 720 days and dissected their respective contribution to the RDOC pool and the underlying mechanisms. Furthermore, we also reveal the responses of *Synechococcus* and heterotrophic bacterial community and their RDOC dynamics under a simulated ocean warming (+4°C) condition. Our study provides novel insights into the dynamics of RDOC genesis and accumulation driven by long‐term algae‐bacteria interactions in the ocean and its fate under ocean warming scenarios.

## 2 Materials and Methods

### 2.1 Establishment of Long‐Term *Synechococcus*‐Bacterial Community Coculture Systems

The axenic *Synechococcus* sp. PCC7002 was obtained from the Pasteur culture collection and cultivated at 22°C under white LED light 12:12 light:dark cycle (50 μmol photons m^−2^ s^−1^). To eliminate the influence of external organic carbon, we used modified SN medium (Waterbury et al. 1986) without EDTA and vitamin B_12_. Prior to the experiment, the axenicity of the *Synechococcus* was verified by agar media and 16S rRNA gene sequencing analysis. A heterotrophic bacterial community was obtained from a previously established *Synechococcus*‐bacteria coexistence system (Zhao et al. 2022). 20 mL of the system was filtered sequentially through sterilized 1.0 and 0.22 μm pore‐size polycarbonate membranes (Millipore, Ireland). The bacterial cells collected on 0.22 μm membranes were gently rinsed three times with autoclaved artificial seawater (ASW) and then resuspended in 2 L of modified SN media containing exponentially growing axenic *Synechococcus* in triplicate cultures. The bacterial and *Synechococcus* initial cell concentrations were ~10^6^ cells mL^−1^ within the coculture systems by flow cytometry.

The coculture systems were cultivated as described above without further addition of any organic and inorganic nutrients. Axenic *Synechococcus* cultures were established following the same protocol but without the addition of any bacteria. The *Synechococcus*‐bacteria coculture systems were maintained for 720 days and samples were collected at days 0, 1, 3, 5, 9, 15, 30, 45, 70, 100, 180, 450, 600, and 720 for measuring the DOC concentration, FDOM, algal and bacterial abundance. Axenic *Synechococcus* cultures were sampled at days 0, 1, 3, 5, 9, 15, 30, 45, 70, and 100 for the above analysis excluding bacterial abundance. Sampling beyond day 100 was not considered, as the culture system was not viable. Additional samples were collected from the coculture system at days 0, 70, 450, and 720 for analyzing the DOC molecular composition and bacterial metagenomics and days 0 and 70 from the axenic system for DOC molecular composition analysis. In addition, another set of *Synechococcus*‐bacteria coculture systems was established for subsequent DOC analysis with 30 days of incubation. DOC accumulation was calculated by subtracting the initial DOC concentration from the subsequent measured DOC samples. The axenic and coculture systems were periodically checked for algal contamination throughout the experimental period as described above.

### 2.2 Determination of DOC Bioavailability Through Long‐Term Microbial Degradation Experiment

The DOC from (i) 720‐day *Synechococcus*‐bacteria coculture systems (referred to as DOC_720‐coculture_), (ii) 30‐day *Synechococcus*‐bacteria coculture systems (DOC_30‐coculture_) and (iii) 70‐day axenic *Synechococcus* cultures (DOC_axenic_) were collected by gentle filtration through 0.22 μm pore‐size membranes (Millipore, Ireland) using a precombusted glass filtration system. The concentration of the collected DOC samples was adjusted to ~8 mg L^−1^ with sterilized ASW (without organic carbon). The microbial community was collected from the surface seawater of the Qingdao coast (36°05′ N, 120°28′ E), China in December 2021. Briefly, the natural seawater was gently vortexed thrice (2 min) and prefiltered sequentially through 20 μm and 1.0 μm pore‐size membranes to remove large particles and detritus. The filtrate was then kept in the dark for 3 days to suppress the growth of autotrophic picoalgae. Subsequently, the filtrate was passed through 0.22 μm membranes to collect microbial cells. The collected microbial communities were suspended in sterilized ASW and added to the adjusted DOC systems in triplicates. These steps also avoid the incorporation of background DOC in seawater into the systems. All systems were covered with 0.22 μm pore‐size permeable membranes and incubated in the dark at 25°C for 150 days. Samples were collected at days 0, 1, 3, 5, 7, 10, 15, 20, 30, 45, 60, 90, 110, 130, and 150 for measuring the DOC concentration. Additional samples were collected at days 0, 7, 30, 90, and 150 for analyzing molecular compositions of DOC.

### 2.3 Collection of DOC Samples From the Western Pacific Ocean

DOC samples were collected in triplicates from the surface (5 m) and deep (2000 m) depths at three distinct sites in the western Pacific Ocean (4.02° S–6.00° N, 162.98° E–163.00° E) during NORC2020‐09 cruise in 2021. Immediately after collection, the seawater samples were filtered through precombusted 0.7 μm GF/F filters and preserved at −20°C until further determination of the DOC molecular composition.

### 2.4 Simulated Ocean Warming Experiment on Long‐Term Coculture System of *Synechococcus* and Bacteria

The long‐term *Synechococcus*‐bacteria coculture systems were kept in separate temperature‐controlled incubators set at 22°C (control) and 26°C (+4°C) for 7 days. The 4°C increase was chosen based on the Intergovernmental Panel on Climate Change (IPCC) predictions of ocean warming, which estimates a temperature increase of 1.1°C–6.4°C over the course of the 21st century (IPCC 2014). The maximum quantum yield of photosystem II (Fv/Fm) ratio of *Synechococcus* on days 0, 3, and 7 was measured by using a dual‐wavelength pulse‐amplitude modulated fluorescence monitoring system (Dual‐PAM, Heinz Walz, Germany) as an indicator of the autotroph's photosynthetic performance in order to determine the growth status of *Synechococcus* (Nair et al. 2022). Samples were collected in the control and treatment coculture systems on days 0, 1, 3, 5, and 7 to measure the DOC concentration, algal and bacterial abundance, and bacterial community composition. Additionally, samples were collected on days 0 and 7 for analyzing the molecular composition of DOC. After the 7‐day incubation period, DOC from the control and treatment coculture systems were collected to investigate their recalcitrance by a long‐term microbial degradation experiment as described above. During the degradation experiment, samples were collected on days 0, 1, 3, 5, 10, 30, 60, 90, and 100 for measuring the DOC concentration.

### 2.5 Analysis of DOC Concentration and FDOM Component

Samples for measuring DOC concentration were acidified at pH 2 with 6 mol L^−1^ hydrochloric acid (HCl) to remove traces of inorganic carbons. Subsequently, the samples were analyzed on a Shimadzu TOC‐VCPH total organic carbon analyzer equipped with an autosampler ASI‐V employing the temperature catalytic oxidation technique (Dai et al. 2012). Fluorescence excitation‐emission matrices (EEMs) were analyzed by using a fluorescence spectrometer (F‐4600, Hitachi high technologies, Japan), parallel factor analysis (PARAFAC) was performed in MATLAB (Mathworks, Natick, MA) with the DOMFluor toolbox (Li et al. 2022).

### 2.6 Solid‐Phase Extraction (SPE) and Analysis of DOC Molecular Composition

Samples for measuring DOC molecular composition were analyzed by solid phase extraction (SPE) coupled with styrene divinylbenzene polymer cartridges (PPL) on a 9.4 Tesla Apex‐ultra FT‐ICR MS (Bruker, Germany) at China University of Petroleum using a standard protocol (He et al. 2020). Due to the limited sample availability in FT‐ICR MS, DOC molecular composition was determined by pooling the three biological replicates into a single sample for analysis. Triplicate samples from coculture, degradation, and warming experiments were combined (approximately 150–450 mL) and were filtered through 0.22 μm polycarbonate membranes. The filtrates were individually acidified in precombusted glass bottles with 6 mol L^−1^ of HCl. Prior to extraction, 15 mL methanol (HPLC‐grade, Sigma‐Aldrich, United States) and 15 mL acidified ultrapure water (pH = 2) were added to the solid phase extraction column (Bond Elut PPL 500 mg cartridges, Agilent, United States) to activate the extraction column (Dittmar et al. 2008). The acidified samples were slowly passed through the activated PPL column to enrich the target compounds. After injection, cartridges were rinsed with acidified ultrapure water (pH = 2) to remove the remaining inorganic salt. Then, the column was dried with high‐purity nitrogen gas and finally eluted with 10 mL of methanol. Extraction efficiency was ∼60% for oceanic DOC. The resulting extracts were analyzed under electrospray ionization negative mode with an infusion rate of 250 μL h^−1^ into the ESI source. The mass spectra of peaks with a signal‐to‐noise ratio > 4 ranged from 200 to 800 m/z and were internally calibrated using data lists of sodium formate and a known mass series in the sample (He, Zhang et al. 2020). Molecular formulas (MFs) were determined using in‐house software (He, Pan et al. 2020) and assigned following the procedure described by Koch and Dittmar (Koch et al. 2007) with maximum elemental abundances of ^12^C_0‐60_^1^H_0‐120_^16^O_0‐3_^14^ N_0‐30_^32^S_0‐2_.

The indexes H/C, O/C, DBE, modified aromatic indices (AI_mod_), and NOSC were calculated with intensity (*M*_i_) and the intensity‐weighted average values (*P*_wa_) (Koch and Dittmar 2006; LaRowe and Van Cappellen 2011; Zhang, Heal et al. 2021). Formulae were categorized into six classes, based on the element ratio and AI_mod_ (Seidel et al. 2015): (1) condensed aromatics (AI_mod_ > 0.66); (2) polyphenol (0.66 ≥ AI_mod_ > 0.5); (3) highly unsaturated and phenolic compounds (AI_mod_ ≤ 0.50 and H/C < 1.5); (4) unsaturated aliphatic *N* = 0 (2.0 > H/C ≥ 1.5); (5) unsaturated aliphatic *N* > 0 (2.0 > H/C ≥ 1.5); and 6) saturated fatty and sulfonic acid (H/C ≥ 1.5 or O/C > 0.9). The CRAM was characterized by the formulas with DBE: C = 0.3–0.68, DBE: H = 0.2–0.95, and DBE: O = 0.77–1.75 (Li et al. 2022). The van Krevelen (VK) diagram was divided into eight regions by plotting the ratios of H/C and O/C for assigned molecular formulas. Each region represented a different category of compounds, included lipids (0 < O/C < 0.3 and 1.5 < H/C < 2), proteins (0.3 < O/C < 0.55 and 1.5 < H/C < 2.2), amino sugars (0.55 < O/C < 0.67 and 1.5 < H/C < 2.2), carbohydrates (0.67 < O/C < 1.2, 1.5 < H/C < 2.2), unsaturated hydrocarbons (0 < O/C < 0.1, 0.7 < H/C < 1.5), CRAM‐like (0.1 < O/C < 0.67 and 0.7 < H/C < 1.5), tannins (0.67 < O/C < 1 and 0.5 < H/C < 1.5), and condensed aromatics (0 < O/C < 0.67 and 0.2 < H/C < 0.7) (He, Zhang et al. 2020).

### 2.7 Classification Criteria for RDOC Molecule

To accurately identify *Synechococcus*‐derived RDOC (RDOC_algae_) and bacterial‐derived RDOC (RDOC_bact_) MFs stringent criteria were employed: RDOC_algae_ MFs were defined as those consistently detected during the 150‐day degradation of both the axenic *Synechococcus* culture DOC (DOC_axenic_) and the *Synechococcus*‐bacteria coculture DOC (DOC_720‐coculture_), as well as throughout the long‐term coculture period. While RDOC_algae_ could be represented simply by the MFs consistently seen during the 150‐day degradation of DOC_axenic_, this more stringent definition was used to better reflect RDOC_algae_ in the coculture systems and avoid overestimation. This was done to minimize the false detection of RDOC MFs that were present throughout the 150‐day degradation of DOC_axenic_ and DOC_720‐coculture_ but not observed consistently throughout the long‐term coculture period. RDOC_bact_ was defined as the total RDOC pool minus MFs consistently seen during the 150‐day degradation of DOC_axenic_. Total RDOC indicates the MFs consistently detected during the 150‐day degradation of DOC_720‐coculture_.

### 2.8 Analysis of Bacterial and *Synechococcus* Abundance, and Bacterial Community Structure

Bacterial and *Synechococcus* abundance was determined by a FACSAria II Flow cytometer (BD Bioscience, United States) following the protocol described previously (Nair et al. 2022). For bacterial community composition, the bacterial cells were collected on a 0.22 μm pore‐size polycarbonate membrane. Total DNA was extracted using the CTAB/SDS DNA extraction protocol. The V3‐V4 variable region of the 16S rRNA gene was amplified with the primer sets 341F (5′‐CCTAYGGGRBGCASCAG‐3′) and 806R (5′‐GGACTACHVGGGTWTCTAAT‐3′). Amplified products were purified using the E.Z.N.A. Gel Extraction Kit (Omega, USA) and sequencing libraries were prepared using the TruSeq DNA PCR‐free library preparation kit (Illumina, USA) following the recommended protocol. Sequencing was performed on an Illumina NovaSeq 6000 platform (Novogene, Beijing, China). The resultant raw sequence reads were demultiplexed and then truncated by trimming the barcodes and primer sequences. Paired‐end reads were processed using the DADA2 pipeline v1.16 (Callahan et al. 2016) in R v4.1 with the following parameters: truncLen = c (220, 220), maxN = 0, maxEE = c (5, 5), truncQ = 2. The amplicon sequences variants (ASVs) were assigned based on sequence similarity thresholds and the taxonomy assignment was determined by aligning the sequences with those in the silva v138.1 database (https://www.arb‐silva.de/documentation/release‐138/). Downstream processing of the bacterial ASVs was carried out using MicrobiomeAnalyst package (Dhariwal et al. 2017) with recommended filtering and normalization.

### 2.9 Metagenomic Sequencing and Analysis of Bacterial Community Function

Total DNA was extracted using the DNeasy PowerSoil Pro Kit (Qiagen, USA) following the manufacturer's instructions. DNA concentration was measured by Qubit3.0 fluorometer (Invitrogen, USA). Metagenome libraries were prepared using Illumina NEB Next Ultra DNA Library Prep Kit (NEB, USA) following the recommended protocol. Libraries were pooled with the cBot Cluster generation system using the Illumina PE cluster kit (Illumina, USA) according to the manufacturer's instructions. Finally, sequencing was performed on Illumina Novaseq 6000 (Novogene, Beijing, China), generating 150‐bp paired‐end reads. Quality assessment of the raw reads was conducted using FastQC (Andrews 2015) v.0.11.9. Adapter removal, quality filtering, and trimming were performed using Trimmomatic (Bolger, Lohse, and Usadel 2014) v.0.3.9 with the following parameters: SLIDINGWINDOW:4:20 MINLEN:50. Host *Synechococcus* reads were filtered out by aligning the cleaned reads against the *Synechococcus* sp. PCC7002 genome (GeneBank accession number GCA_000019485.1) using Bowtie (Langmead and Salzberg 2012) v.2.5.1 and samtools (Danecek et al. 2021) v.1.6. The high‐quality forward and reverse reads were co‐assembled into contigs using metaSPAdes (Prjibelski et al. 2020) v.3.11.1 with the k‐mer (‐k) option as 21, 33, 55, 77, 99, and 127. Assembly statistics were generated using quast (Mikheenko, Saveliev, and Gurevich 2016) v.5.0.2. The protein‐coding genes from the assembled contigs were predicted using Prodigal (Hyatt et al. 2010) v.2.6.3 with default parameters and a non‐redundant set of genes was obtained with CD‐HIT v4.8.1 (‐c 0.95, ‐G 0, ‐aS 0.9, ‐g 1, and ‐M 0). The gene abundances in each sample were normalized to reads per kilobase per million mapped reads (RPKM) using CoverM (Ben 2022) v0.6.1 (‐min‐read‐percent‐identity to 95, ‐min‐read‐aligned‐percent to 75, and –methods rpkm), which is commonly used in metagenomics to counteract the effects of gene lengths and sequencing depths. Functional annotation was carried out using eggNOG‐mapper (Cantalapiedra et al. 2021) v2.1.10 and eggNOG orthologous groups database (Huerta‐Cepas et al. 2019) v5.0 with an *e*‐value cutoff of 1e–8. Differential abundance of KEGG orthologs (KOs) between day 0 and 720 of long‐term cocultures was determined with DESeq2 (Love, Huber, and Anders 2014) v. 1.28.1 with a false discovery rate (FDR) adjusted *p*‐value (*p*‐adj) of < 0.05. Additionally, the secondary metabolite biosynthetic gene clusters (BGCs) in each contigs were annotated using antiSMASH (Blin et al. 2023) v7.0 with default parameters, clustered into gene cluster families (GCFs) using BiG‐SCAPE (Navarro‐Munoz et al. 2020) v1.1.2 and their abundance was calculated in RPKM using BiG‐MAP (Pascal Andreu et al. 2021).

In addition, the assembled contigs were binned using the MetaWRAP (Uritskiy, DiRuggiero, and Taylor 2018) v.1.3.2 pipeline to obtain a set of non‐redundant MAGs. CheckM (Parks et al. 2015) v1.1.3 was employed to evaluate the quality of the MAGs and those MAGs with > 50% completeness with < 10% contamination were considered for downstream analysis. This generated 44 high quality (> 90% completeness) and 5 medium‐quality (> 60% completeness) MAGs. The taxonomic identity of the 49 MAGs was determined using the Genome taxonomy database toolkit (GTDB‐Tk) (Chaumeil et al. 2020) v1.5.0 employing the GTDB database (Parks et al. 2022) vr22. A phylogenetic tree was constructed through IQ‐TREE (Minh et al. 2020) v2.0 with the MFP evolutionary model and the ultrafast bootstrapping value of 1000 based on multiple sequence alignment file generated by GTDB‐Tk. The tree was visualized using iTOL v6.1.2. Furthermore, the abundance of MAGs was calculated using the metaWRAP Quant bins module. Finally, the metabolic potential of MAGs was predicted by KEGG‐Decoder v1.0.8.2 (www.github.com/bjtully/BioData/tree/master/KEGGDecoder).

### 2.10 Structure Prediction of RDOC Molecules and Their Potentially Related Genes

The RDOC_bact_ MFs were searched against the PubChem chemical database (https://pubchem.ncbi.nlm.nih.gov/search/search.cgi) based on the structure pattern (number and type of atomic elements) using default settings. The resultant compound structures were then screened against the KEGG compound (https://www.genome.jp/kegg/), BioCyc (https://www.biocyc.org/), and the Natural Products Atlas (van Santen et al. 2022) (https://www.npatlas.org/) databases to remove matches with synthetic origin. The remaining potential natural compound structures were then taxonomically classified using ClassyFire (Djoumbou Feunang et al. 2016) (http://classyfire.wishartlab.com) with ChemOnt v.2.1 (Djoumbou Feunang et al. 2016) databases (The detailed process is provided in Data S1). The enzyme commission (EC) numbers of compounds obtained from KEGG and BioCyc were manually matched with the EC numbers of predicted KEGG annotated genes from the metagenomic functional analyses. RDOC_algae_ MFs annotations were collected through previously published *Synechococcus* sp. PCC7002 metabolite data (Baran et al. 2010; Baran, Bowen, and Northen 2011; Baran et al. 2013; Bennette, Eng, and Dismukes 2011; Jones et al. 2021). To identify the potential genes related to the RDOC_algae_ MFs, a *Synechococcus* sp. PCC7002 MAG was obtained from the metagenome of the long‐term coculture system. In brief, the prefiltered host *Synechococcus* sp. PCC7002 reads were assembled separately as described above. Magpurify v2.1 (Nayfach et al. 2019) and CheckM were used to remove contamination and assess the quality of the assembled MAG. The MAG was determined to be a high‐quality draft MAG (100% completion, with 0.76% contamination) according to the Minimum Information about a MAG (MIMAG) (Bowers et al. 2018). Gene prediction, gene abundance quantification and functional annotation were performed as described above. We utilized *Synechococcus* PCC7002 MAG rather than the published PCC7002 genome, as the complete MAG more likely provides a more comprehensive representation of algal adaptations and genomic variations under our long‐term coculture conditions.

### 2.11 Determination of Inorganic *N* and *P* and Other Environmental Parameters

Concentrations of inorganic nutrients (NO_3_^−^, NO_2_^−^, NH_4_^+^, and PO_4_^3−^) in the initial and final stages of the coculture experiment system were analyzed using an AutoAnalyzer (BRAN and LUEBBE AA3, Germany) following standard methods (Chen et al. 2020). Dissolved oxygen saturation and pH were determined using a calibrated fiber‐optic multi‐meter (Pyro Science, Germany) from the initial and final stages of the coculture system.

### 2.12 Statistical Analysis

All the data generated in this study are available in figshare (Zhao et al. 2024). DOC decay rates were estimated using a first‐order decay model based on the multi‐G model (Guillemette and del Giorgio 2011), where DOC_labile_ is the bioavailable fraction of the DOC, DOC_residual_ is the residual fraction of the DOC, k is the first‐order decay constant and t is the time required for degradation. All experiments were performed in triplicate except as otherwise noted, and the results were presented as mean ± standard deviation. Statistical analysis was performed using Prism 8 (GraphPad Software, USA) and significant differences were determined at *p* < 0.05. The significance between the DOC concentrations of the coculture system and the axenic culture system in the first 100 days was calculated using an unpaired t‐test. Mann–Whitney *U* test was used to determine the significance between first‐order decay constants of each sample. Moreover, Mann–Whitney U test was also used to evaluate the differences in DOC concentration at the initial and final stages of degradation, as well as the differences in degradation amount between the treatment group and the control group in the warming degradation experiment. Two‐way ANOVA and multiple comparison test analysis were applied to compare the significant changes in Fv/Fm and DOC values over time between two temperature treatments.

## 3 Results

### 3.1 Long‐Term *Synechococcus*‐Bacteria Interactions Lead to a Gradual Accumulation of DOC in Seawater to Exceptionally High Concentrations

**FIGURE 1:**
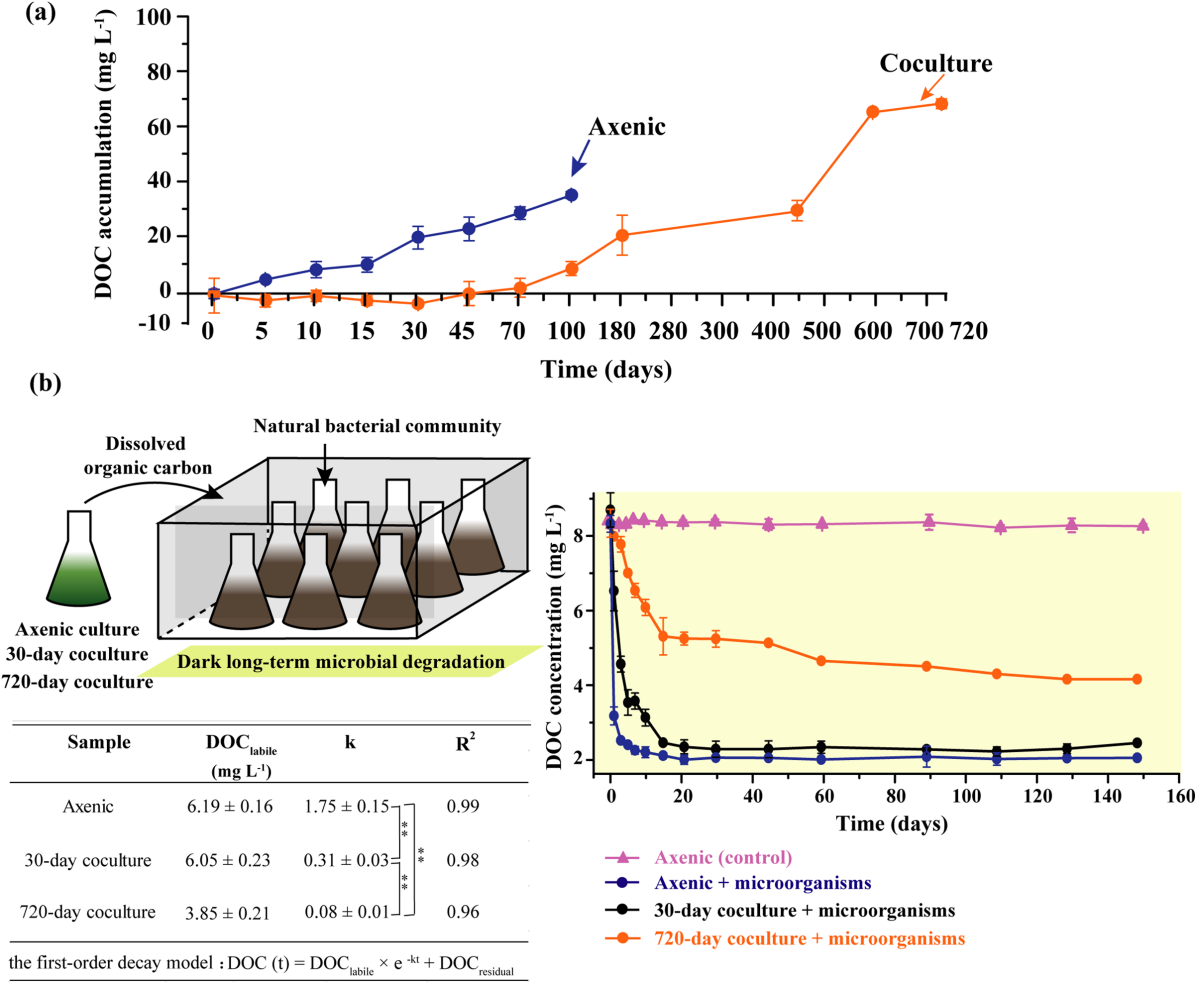
Dynamics of DOC in long‐term *Synechococcus*‐bacteria coculture and axenic cultures systems. (a) Variations in DOC accumulation over time in axenic *Synechococcus* cultures (blue) and *Synechococcus*‐bacteria cocultures (orange). Error bars indicate standard deviation (SD, *n* = 3). (b) Long‐term microbial degradation of DOC from axenic cultures and cocultures in dark incubations. The first‐order decay model was used to estimate the decay rate of DOC. DOC_labile_ represents the bioavailable fraction of DOC, DOC_residual_ represents the residual fraction of DOC, *k* is the first‐order decay constant, *t* is the time required for degradation, and *R* ^2^ represents the coefficient of determination. An asterisk (*) indicates a significant difference using the Mann–Whitney *U* test, and ** denotes *p* < 0.01.

Here, we constructed a long‐term algae‐bacteria mutualistic model system. The *Synechococcus* and the bacterial community exhibited similar growth dynamics as previously reported (Nair et al. 2022) and maintained survival for 720 days (Data S1). During the initial 100 days, the bacterial abundance in the *Synechococcus*‐bacteria coculture systems increased continuously (8 × 10^6^ cells mL^−1^ at day 100), while the abundance of *Synechococcus* initially increased and then gradually declined (3 × 10^5^ cells mL^−1^ at day 100). The DOC concentration in the coculture systems was 2.5‐fold lower (24.41 ± 2.9 mg L^−1^) compared to the axenic *Synechococcus* culture systems (51.15 ± 1.7 mg L^−1^; unpaired *t*‐test, *p*‐adj < 0.05). The DOC accumulation during the initial 100 days within the *Synechococcus*‐bacteria coculture systems was over 3‐fold lower (9.8 ± 1.8 mg L^−1^) in comparison to the axenic *Synechococcus* culture systems (36.0 ± 1.0 mg L^−1^; Figure 1a), likely due to bacterial consumption of organic carbon (Data S1). However, after 600 days, DOC gradually increased to a concentration as high as 69.7 ± 1.2 mg L^−1^ in *Synechococcus*‐bacteria coculture systems. In contrast, all axenic *Synechococcus* cultures died by 100 days, and the final DOC accumulation was below 36.0 mg L^−1^ in the axenic system. While lower than the long‐term cocultures, these axenic DOC levels were still notable. However, this DOC was largely comprised of bioavailable LDOC, as determined through the degradation experiments in this study and previous studies (Wang et al. 2022; Zhao et al. 2019; Zheng et al. 2020). The DOC concentrations and other data for axenic culture were not collected beyond 100 days, as the culture system was not viable.

### 3.2 Gradual Increase in the Recalcitrant Fraction of DOC (RDOC) During the Long‐Term *Synechococcus*‐Bacteria Cocultivation

Furthermore, we examined the bioavailability of the accumulated DOC in the 720‐day long‐term *Synechococcus*‐bacteria cocultures (DOC_720‐coculture_) through a 150‐day microbial degradation experiment using natural seawater microbial communities (Figure 1b). Simultaneously, we monitored the same in the 70‐day old axenic culture (DOC_axenic_) and 30‐day old (DOC_30‐coculture_) coculture systems. To preclude interference from exogenous DOC molecules, we conducted the experiment in artificial seawater without organic carbon. The degradation pattern was well described by a first‐order decay model (*R*_2_ = 0.95–0.99). The model revealed that out of the 8.34 ± 0.37 mg L^−1^ of the initial DOC proportion, only 3.85 ± 0.21 mg L^−1^ (DOC_labile_) could be degraded by the natural bacterial community in the DOC_720‐coculture_, equivalent to 46% labile DOC (Figure 1b). The remaining 54% of the DOC_720‐coculture_ persisted throughout the 150‐day experimental period, suggesting resistance to microbial degradation. In contrast, the degradable fractions of DOC_axenic_ and DOC_30‐coculture_ were higher, with DOC_labile_ values of 6.19 ± 0.16 and 6.05 ± 0.23 mg L^−1^, respectively, equivalent to 71%–74% labile DOC. Additionally, the decay constant (*k*) for DOC_720‐coculture_ was 0.08 day^−1^, substantially lower than k values for DOC_axenic_ (1.75 day^−1^) and DOC_30‐coculture_ (0.31 day^−1^). The markedly slower k indicates greater recalcitrance of the DOC in the long‐term cocultures compared to that in the axenic cultures and 30‐day cocultures.

### 3.3 Long‐Term *Synechococcus*‐Bacteria Interaction Greatly Enhanced DOC Molecular Diversity and Recalcitrance

**FIGURE 2:**
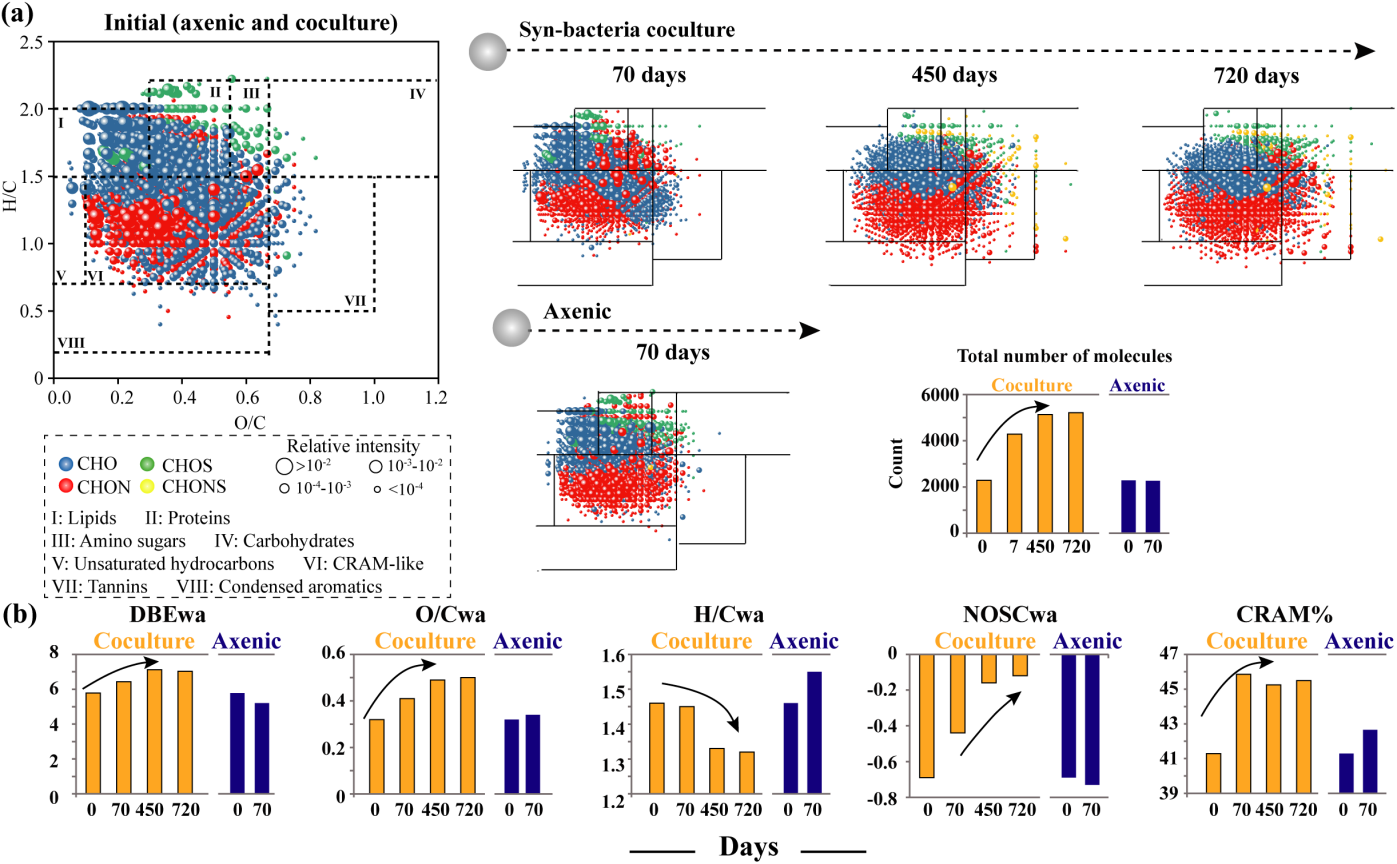
Molecular properties of DOC in axenic *Synechococcus* cultures and long‐term *Synechococcus*‐bacteria coculture systems. (a) Van Krevelen diagrams of DOC molecular formulas in different incubation time points (*Synechococcus*‐bacterial cocultures: Initial, 70, 450, and 720 days; axenic *Synechococcus* cultures: Initial and 70 days). H/C and O/C represent the hydrogen‐to‐carbon ratio and oxygen‐to‐carbon ratio, respectively. The dotted lines in the Van‐Krevelen diagram correspond to major classes of compounds included in regions 1–8: Lipids, proteins, amino sugars, carbohydrates, unsaturated hydrocarbons, CRAM‐like, tannins, and condensed aromatics, respectively. The circle color represents compound categories and size represents relative intensities. Bar graph displays the total number of molecules. (b) Variations in molecular characteristic indexes of DOC in the axenic and coculture systems on days 0, 70, 450, and 720. wa represents weighted average values, DBE represents double bond equivalents, NOSC represents the nominal oxidation state of carbon and CRAM represents carboxyl‐rich alicyclic molecule‐like organic matter.

Characterization of the solid phase‐extracted DOC (PPL‐DOC) molecular formulas (MFs), revealed an obvious enhancement in both the molecule diversity and recalcitrance nature of DOC during the long‐term coculture process. Overall, the coculture system exhibited a higher number of distinct DOC MFs than the axenic culture systems. In the coculture system, the number of MFs increased substantially from an initial count of 2291–5208 by day 720 (Figure 2a; Data S1). In contrast, the abundance of MFs remained relatively stable in the axenic systems.

Furthermore, the characteristic indices of these MFs were calculated to evaluate the degree of their recalcitrance (Li et al. 2022). The weighted average values (wa) of double bond equivalents (DBE), oxygen‐to‐carbon ratio (O/C), and the nominal oxidation state of carbon (NOSC), displayed higher values during the later stages (days 450 and 720) compared to the early stages (day 0 and 70) in the coculture system (Figure 2b). Concurrently, the wa values of the hydrogen‐to‐carbon ratio (H/C), which typically is lower for RDOC (Hu et al. 2023), decreased below a value of 1.4. This suggests an increase in DOC bio‐recalcitrance during the cocultivation progress. In contrast, the axenic system exhibited minimal changes in the DBE, O/C, and NOSC values, while the H/C value increased above 1.5. CRAM represents a class of potentially recalcitrant components (Li et al. 2022). The percentage of CRAM components exhibited a marked increase of 4.4% in the long‐term cocultivation systems, whereas the axenic systems displayed a modest increase of just 1.4% (Figure 2b), indicating long‐term *Synechococcus*‐bacteria interaction enhanced DOC recalcitrance.

### 3.4 Bacterial Community Activity Generates Diverse RDOC Molecules

**FIGURE 3:**
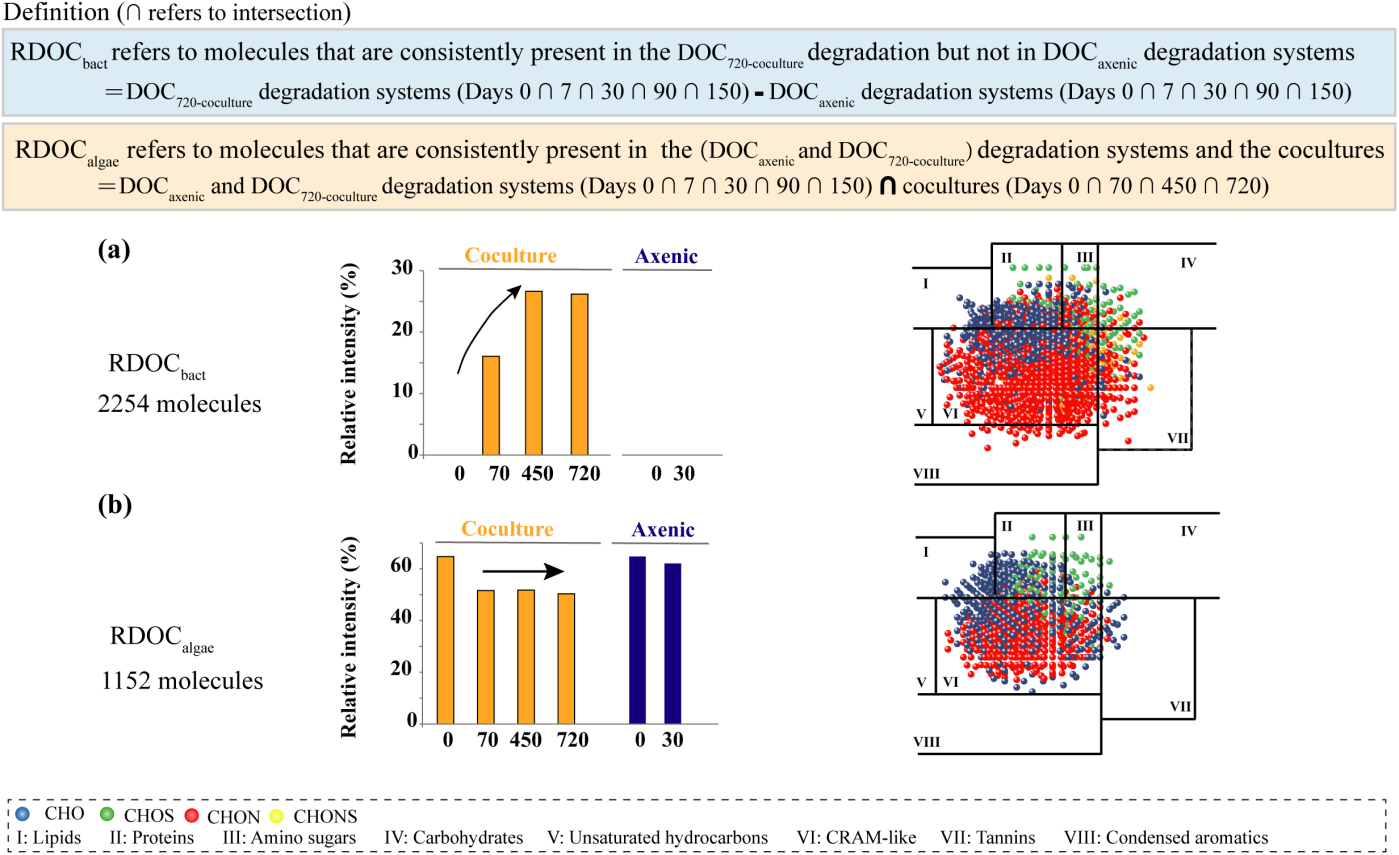
Compositional changes in RDOC molecules produced by *Synechococcus* and bacteria in long‐term cocultures. Variations in relative intensity (%) of RDOC molecules derived from (a) bacteria (RDOC_bact_) and (b) *Synechococcus* (RDOC_algae_) and over time. RDOC_bact_ represents molecules that are only present in degradation systems of cocultures and not in axenic degradation systems. RDOC_algae_ represents DOC molecules consistently present in degradation systems of axenic cultures and cocultures, as well as during the long‐term coculture period. The corresponding Van Krevelen diagrams display the contribution of different molecular formulas to RDOC_bact_ and RDOC_algae_ DOC pool.

Heterotrophic bacteria are recognized as a major contributor to marine RDOC genesis. By employing stringent identification criteria (Figure 3), we delineated 2254 RDOC MFs associated specifically with bacteria. These were termed RDOC_bact_ MFs (Figure 3a; Data S1). The relative intensity of the identified RDOC_bact_ MFs showed a clear increase during the long‐term cocultivation. Starting from none at day 0, the peak intensity of these molecules reached 16% by day 70 and further increased to 26% by day 720. This indicates a substantial accumulation of complex and diverse RDOC molecules by bacterial communities in long‐term coculture systems.

In terms of molecular composition, RDOC_bact_ included MFs from lipids, proteins, amino sugars, carbohydrates, CRAM‐like, and tannins classes. Nitrogen and sulfur‐rich molecules accounted for 60.2% of the total molecular composition. To gain further insight into these molecules, a total of 536 MFs from RDOC_bact_ were structurally predicted and grouped into more than 20 broad categories (Data S1) by comparing these MFs with public databases, reflecting the diversity of RDOC_bact_. These included diverse complex cyclic, aromatic and substituted structures such as polycyclic aromatic hydrocarbons (PAHs: benzopyrans, naphthalenes), steroids derivatives (oxosteroids, steroid esters), alkaloids (phenanthrols, piperidinones), benzenoid derivatives (benzenediols, benzodiazines), flavonoids (flavonoid glycosides, o‐methylated flavonoids), quinones (naphthoquinones, anthraquinones), terpenoids (diterpenoids, sesquiterpenoids), carboxylic acids conjugates, and others. Intriguingly, most of these predicted compounds were conjugates of other diverse compounds, containing rigid aromatic ring structures and hydrophobic side chains that likely hindered their further catabolism. Their consistent presence throughout the degradation experiment and the substantial increase in their recalcitrance signatures (Figure 2b), corroborate the inherent recalcitrant nature of RDOC_bact_.

### 3.5 Genetic Potentials for the Production and Accumulation of RDOC Molecules by Bacterial Communities in Long‐Term Co‐Culture Systems

The increasing proportion of DOC molecular diversity during the long‐term cocultivations may provide greater niche space, enabling increased diversity and abundance of bacterial functional genes (Logue et al. 2016). Here, a differential gene abundance analysis (*p*‐adj < 0.05) of the metagenomes from the long‐term coculture system demonstrated that the bacterial community significantly directed their metabolism towards complex DOC synthesis and degradation during the later stages (days 450–720) of the coculture system (Data S1). Notably, gene orthologs of pathways for steroid, styrene, aminoglycan, and polycyclic aromatic hydrocarbons (PAHs) degradation were significantly abundant at day 720 in comparison to day 0. Most of these genes showed a substantial increase in their abundance after day 70, implying a continuous enrichment of these compounds within the coculture system. Intriguingly, the genes regulating the biosynthesis of complex secondary metabolites such as polyketides, carotenoids, ubiquinone, terpenoids, and alkaloids were also significantly abundant at day 720. Among these, terpene, beta‐lactone, and hserlactone biosynthetic gene clusters (BGCs) were highly represented (Data S1), as were clusters for non‐ribosomal peptides (NRPS), type I polyketides (TIPKS), and type III polyketides (T3PKS). The enrichment of these BGCs, which generate hydrophobic compounds like terpenoids, aromatics, and peptides, suggests a genetic capacity of the bacterial community to synthesize complex RDOC metabolites. Additionally, metagenome‐assembled genomes (MAGs) obtained from the coculture system exhibited abundant genes encoding the Methyl Erythritol Phosphate/1‐deoxy‐D‐xylulose‐5‐phosphate (MEP‐DOXP) pathway, which is essential for the synthesis of terpenoids and polyketides (Data S1). This may contribute to the accumulation of CRAM molecules in the system, as small molecules tend to form precursors to CRAM analogs via the MEP‐DOXP pathway.

**FIGURE 4:**
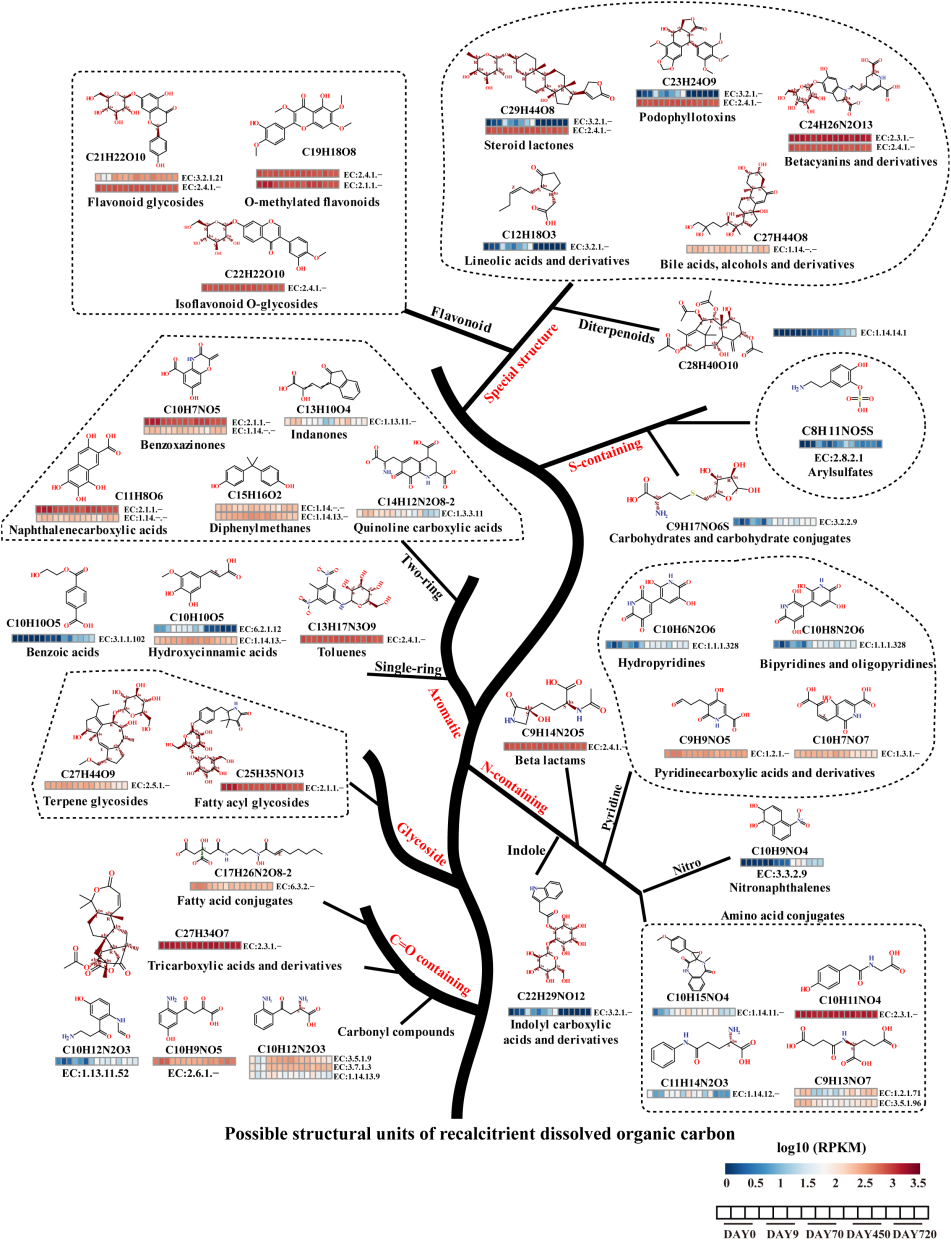
Predicted RDOC structures and associated microbial enzymes. Structures of 31 putative RDOC molecules identified in the microbial metagenome and their matched enzyme commission (EC) numbers involved in RDOC synthesis or transformation. Heatmaps below each structure depict the log10‐transformed RPKM‐normalized abundance of predicted genes for each EC number over the coculture period.

Moreover, we correlated the predicted functional genes with the predicted RDOC_bact_ molecules based on corresponding enzyme commission numbers (Figure 4; Data S1). This revealed 31 RDOC_bact_ compounds including pyridine carboxylic acids, indolyl carboxylic acids, hydroxycinnamic acids, flavonoids, terpenoids, steroids, and heterocyclic aromatics. Our analysis suggested that enzyme families such as monooxygenase, dioxygenase, dehydrogenase, glycosidase, glycosyltransferase, and methyltransferase may be involved in the synthesis, utilization, and modification of these compounds. However, the specific activities and interactions of these enzymes, as well as their actual roles in the formation of RDOC, remain unknown. Collectively, these findings indicate that the bacterial community in the long‐term coculture systems contributes to the production and accumulation of RDOC through the synthesis and modification of DOC compounds.

## 4 *Synechococcus* Could Directly Contribute to RDOC Genesis

We tracked the changes in DOC components by analyzing fluorescence dissolved organic matter (FDOM) data in long‐term coculture and axenic culture systems (Data S1). Strikingly, we observed consistent accumulation of a humic‐like FDOM component (C4) in the long‐term coculture systems as well as in the axenic culture system, suggesting a potential direct release of RDOC by the algae. The gradual accumulation of component C4 despite bacterial activity in the coculture system and normal irradiance (50 μmol photons m^−2^ s^−1^) further supported this. Moreover, humic‐like components released by *Synechococcus* were previously hinted to be resistant to bacterial degradation (Zhao et al. 2017). Subsequently, through stringent criteria (Figure 3), we identified distinct 1152 MFs associated specifically with *Synechococcus*‐derived RDOC (RDOC_algae_) (Figure 3b; Data S1), providing robust evidence that the *Synechococcus* could directly contribute to the RDOC genesis. Notably, the relative peak intensity exhibited by RDOC_algae_ MFs was 50%–65%, showing the important contribution of RDOC_algae_ in the accumulated RDOC of the long‐term coculture system. In terms of molecular composition, RDOC_algae_ contained lipids, proteins, amino sugars, carbohydrates, CRAM‐like, and tannins molecules, similar to RDOC_bact_. However, RDOC_algae_ contributed less CRAM‐like, tannins and condensed aromatics molecules than RDOC_bact_ (Figure 3b). To gain further insight into these molecules, we predicted 23 specific compounds by comparing the above MFs with those from the metabolomic datasets of cyanobacteria containing *Synechococcus* sp. PCC7002. These compounds mainly consisted of conjugates of amino acids (gamma‐aminobutyric acid (GABA)‐linked mycosporine), amino sugars (anhydrous muramic acid), cyclic lactones (tricholactone, gloeolactone and sacrolide A), quinolones (hydroxyquinoline, kalkipyrones and yoshinones), carotenoid (palythene) and other compounds (Data S1).

Furthermore, analysis of *Synechococcus* sp. PCC7002 high‐quality draft MAG assembled from the metagenome of the long‐term culture system revealed distinct genes encoding enzymes involved in potential biosynthesis pathways of RDOC_algae_ molecules (Data S1). Specifically, the presence of multiple *mur* genes (*murA*, *B*, *C*, *D*, *E*, *F*, and *G*) points to the capacity for muramic acid production. Genes encoding 3‐dehydroquinate synthase (*aroB*, *aroQ*) and epsilon‐lactone hydrolase (*mlhB*, *chnC*) suggest capabilities for synthesizing aromatic amino acid such as mycosporine conjugates and cleaving lactone rings to form lactone derivatives (Llewellyn et al. 2020), respectively. Moreover, the PCC7002 MAG also carried multiple carotenoid biosynthesis genes (*crtA*, *B*, *E*, *H*, *P*, *R*, *U*, *W* and *ISO*). The identification of this genetic potential, along with the long‐term prevalence of the related compounds in the coculture system, provides evidence that *Synechococcus* sp. PCC7002 can directly contribute to the marine RDOC pool.

## 5 RDOC Species in Long‐Term *Synechococcus*‐Bacteria Interactions Are Similar to Those Found in Oceanic RDOC Pools

**FIGURE 5:**
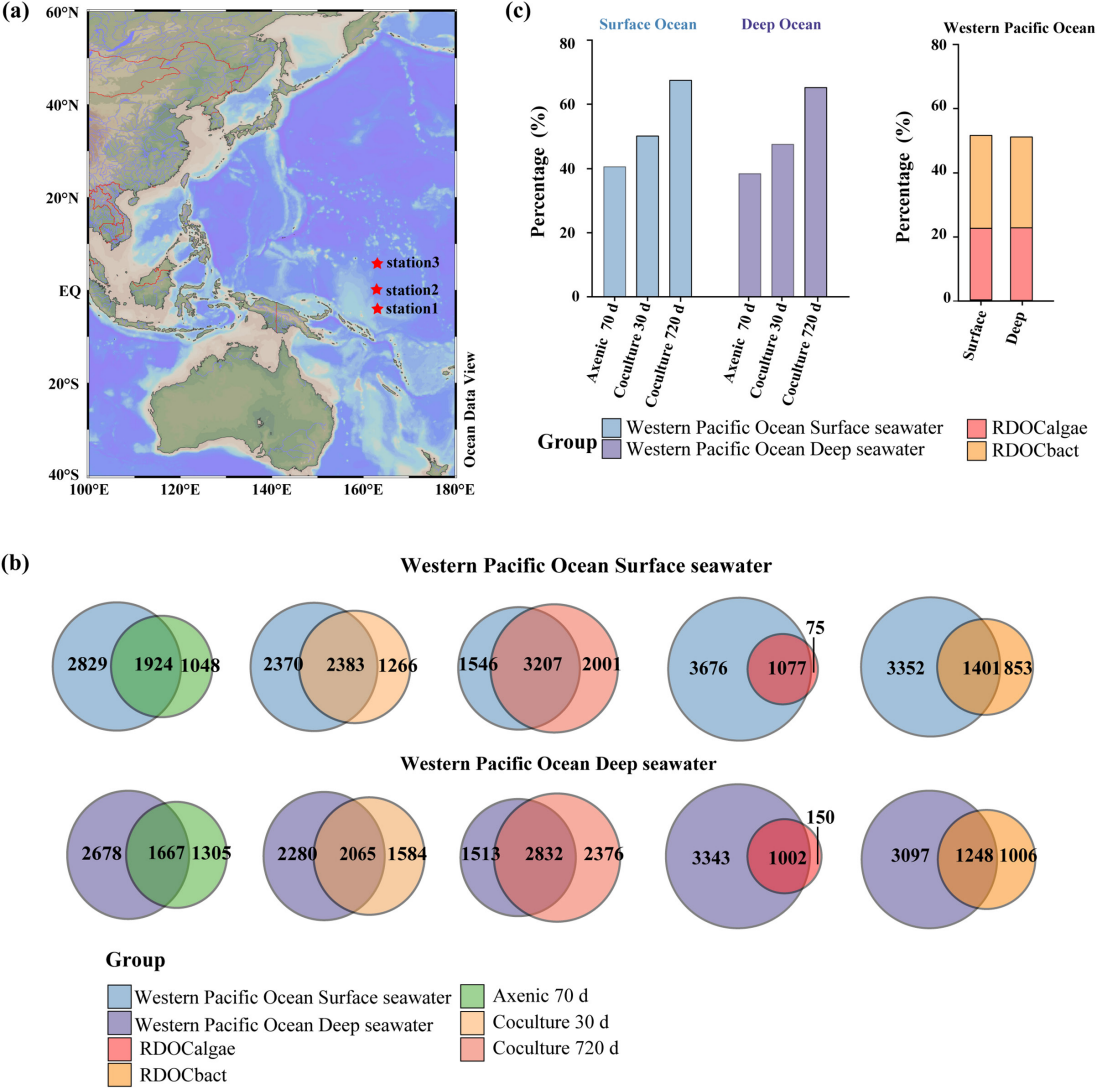
Comparison between molecular formulas (MFs) of DOC from long‐term *Synechococcus*‐bacteria coculture systems to surface and deep Pacific Ocean DOC MFs. (a) Map depicting the location in the western Pacific Ocean where samples were collected. (b) Venn diagrams showing the shared and unique DOC MFs between 70‐day axenic *Synechococcus* cultures, 30‐day cocultures, 720‐day cocultures, and surface and deep western Pacific Ocean samples. (c) Left bar graph displays the percentage of molecular formulae shared between different culture systems and in situ ocean samples. Right bar graph illustrates the proportion of RDOC_algae_ and RDOC_bact_ molecular formulae shared with the molecular formula of the in situ ocean samples. Map lines delineate study areas and do not necessarily depict accepted national boundaries.

Intriguingly, a detailed comparison of the PPL‐DOC derived MFs from our experimental study with that of the in situ surface (5 m) and deep (2000 m) western Pacific Ocean revealed notable consistencies (Figure 5; Data S1). Approximately 67% of the surface ocean PPL‐DOC MFs shared similarities with that of the long‐term *Synechococcus*‐bacteria interactions, while 50% were comparable to short‐term (30‐day) interactions and 40% to the axenic *Synechococcus* cultures. Similarly, the PPL‐DOC MFs from the long‐term *Synechococcus*‐bacteria coculture systems accounted for ~65% of the MFs identified in the deep sea PPL‐DOC, compared to 47% similarity from the short‐term interactions and less than 40% from the axenic *Synechococcus*. Remarkably, RDOC MFs formed during the long‐term coexistence of *Synechococcus* and bacteria contributed over 50% of the surface and deep ocean PPL‐DOC MFs, of which 22.7%–23.1% were *Synechococcus*‐derived (RDOC_algae_) and 28.7%–29.5% were from heterotrophic bacteria (RDOC_bact_). Collectively, these results suggest that long‐term marine algae‐bacteria interactions may substantially contribute to ocean PPL‐DOC pools, with the majority of them being recalcitrant molecules. However, further validation with data from additional sampling sites and diverse oceanic areas is necessary to fully extend these findings to the global ocean RDOC pool.

## 6 Ocean Warming Promoted the Growth of Algae and Bacteria, and Increased DOC Concentration, but Reduced the Proportion and Concentration of RDOC

**FIGURE 6:**
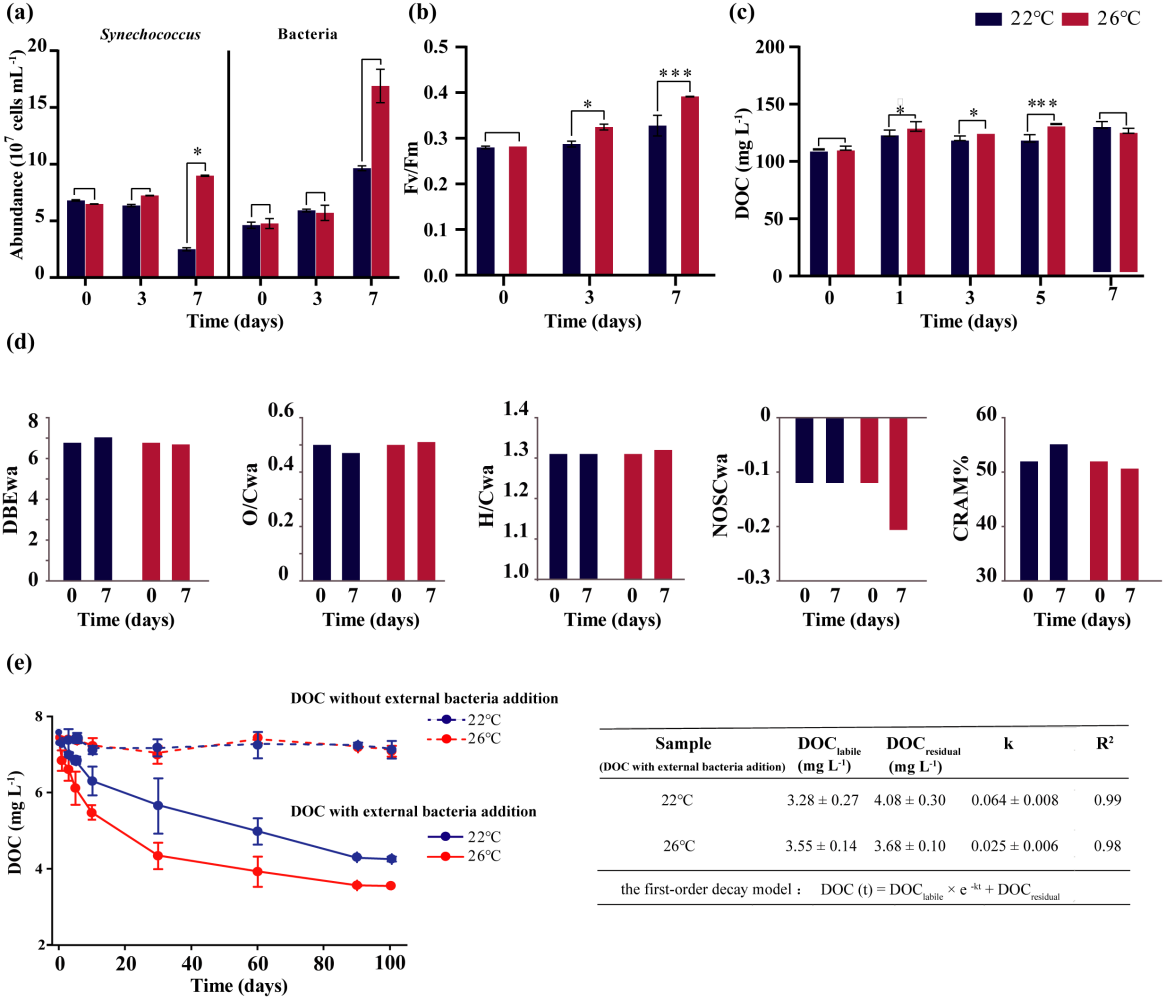
Effects of elevated temperature on *Synechococcus*‐bacteria cocultures and associated DOC dynamics. Variations in (a) *Synechococcus* and bacterial cell abundance, (b) maximum quantum efficiency of photosystem II (Fv/Fm), and (c) DOC concentration, at 22°C and 26°C. Error bars indicate standard deviation (SD); asterisks indicate significant differences between temperatures (two‐way ANOVA and t‐tests; *p < 0.05, ***p < 0.001). (d) Changes in DOC molecular indices (DBEwa, O/Cwa, H/Cwa, NOSCwa, CRAM%) at 22°C and 26°C. (e) Long‐term microbial degradation of DOC from cocultures incubated at different temperatures in the dark compared to cell‐free controls. The first‐order decay model estimated the decay rate of DOC. DOC_labile_ is the bioavailable fraction, DOC_residual_ is the residual fraction, *k* is the decay constant, *t* is the time for degradation, and *R* ^2^ is the coefficient of determination.

The long‐term *Synechococcus*‐bacteria coculture system was cultured for 7 days under simulated ocean warming (+4°C) conditions, the growth of *Synechococcus* and the bacterial community were strongly enhanced as evidenced by a 3.8‐fold (*p‐*adj = 0.03) and 1.7‐fold increase in *Synechococcus* and bacterial abundances, respectively (Figure 6a). The photosynthetic performance (maximum quantum efficiency of photosystem II, Fv/Fm) of *Synechococcus* also significantly increased (*p*‐adj = < 0.01) (Figure 6b). As expected, the DOC concentrations were also elevated significantly (*p‐adj* = < 0.03) for the first 5 days of exposure compared to control cultures at ambient temperature (Figure 6c). However, the difference in DOC between elevated and control temperature conditions was no longer significant (*p*‐adj = < 0.058) in the later period of exposure (Figure 6c), potentially due to enhanced bacterial consumption. Nonetheless, we observed that warming had a clear effect on the properties and molecular composition of DOC. For instance, the 7‐day exposure resulted in a decrease in the proportion of the NOSCwa and CRAM% indices (Figure 6d). Additionally, the elevated temperature did not significantly affect bacterial community composition (Data S1).

Furthermore, we inoculated a natural microbial community into the DOC collected after 7 days from the control and warming treatments and conducted a 100‐day DOC degradation experiment. Fitting a first‐order decay model, it was found that the biodegradable labile DOC (DOC_labile_) was 3.54 ± 0.14 mg L^−1^ in the warming group (47.6%) and 3.28 ± 0.27 mg L^−1^ (42.3%) for the control group. The degradation rate constant (*k*) was 0.43 ± 0.006 day^−1^ for the control group versus 0.52 ± 0.005 day^−1^ for the warming group. Due to the consistent initial concentration settings for the degradation experiments, there is no difference between the two groups in the early stage of degradation, but significant differences emerge in the final stages (days 90 and 100) and in the degradation amount (Mann–Whitney *U* test, *p*‐adj < 0.05). Accordingly, the concentration of RDOC was reduced to 59.0 mg L^−1^ in the warming group compared to 72.8 mg L^−1^ in the control group (Figure 6e). Overall, these results provide evidence that warming indirectly reduced the proportion and concentration of the RDOC molecules by transforming it towards more biodegradable forms.

## 7 Discussion

Our study provides novel insights into the dynamics of RDOC accumulation driven by long‐term algae‐bacteria interactions in the oceans and the fate of their RDOC dynamics under climate change. We demonstrate that the long‐term continuous metabolic interaction between living algae, *Synechococcus* and associated heterotrophic bacterial community leads to the gradual accumulation of DOC to exceptionally high concentrations (69.7 ± 1.2 mg L^−1^), up to two orders of magnitude (~80‐fold) higher than the average concentration in modern pelagic seawater (Hansell 2013). Notably, such high DOC concentrations of two orders of magnitude larger than that observed in modern oceans have been predicted to exist in the Proterozoic oceans (Rothman, Hayes, and Summons 2003). Coincidently, autotrophic cyanobacteria were highly abundant and were the major primary producers in Proterozoic oceans (Hurley et al. 2021). Their primary production activity and active interactions with associated microbiota (Rothman, Hayes, and Summons 2003) may have also contributed to the high DOC levels, through direct release or via microbial pump activity (Jiao et al. 2014). In this context, we offer novel biological perspectives on the accumulation of RDOC during this geological period, complementing the known physical factors such as stratification (Jiao et al. 2024). Indeed, geological insights from black shales (sedimentary rocks containing high organic carbon content) dating to the Proterozoic Ocean suggest a dominant contribution from autotrophic cyanobacteria to the organic carbon burial (Hurley et al. 2021). Our findings provide empirical evidence of long‐term, continuous algae‐bacteria interactions to generate exceptionally high concentrations of DOC, although external biotic and abiotic factors may further limit DOC production and accumulation. Interestingly, throughout the experimental period, the algal and bacterial abundances averaged 10^6^ to 10^7^ cells mL^−1^, which is comparable to average abundances observed in organic matter‐rich oceanic regions (Flombaum et al. 2013; Kim 2019; Li 1998), implying that even conservative estimates of per cell DOC generation could contribute substantially to oceanic DOC inventories. It is noteworthy that the accumulation of DOC driven by algae and bacteria remains limited over the long term, despite the actively growing *Synechococcus* cells. This is evident from the slower DOC accumulation during the later stages of *Synechococcus*‐bacterial cocultivation. Empirically, the high abundance of *Synechococcus* cells should result in a substantial increase in DOC by exudation, even if the bacteria consume most of it. The potential reasons may be: (1) The elevated DOC concentration might impede the additional secretion of DOC by algal cells, especially since some DOC may be released passively by algae, which is easily limited by high extracellular DOC concentrations (Christie‐Oleza et al. 2017). (2) Newly released DOC from algae often stimulates bacterial activity, causing the degradation and conversion of existing DOC molecules through the priming effect (Zhang et al. 2023). (3) Certain concentration‐dependent RDOC (RDOCc) may start to decompose as their concentrations rise, due to the addition of similar DOC molecules (Lennartz and Dittmar 2022).

More importantly, the accumulated DOC in the long‐term cocultures became increasingly recalcitrant, with the proportion of stable RDOC reaching 54%. This percentage was comparable to the RDOC proportion in the surface ocean (Ding et al. 2022; LaBrie et al. 2022; Pedler, Aluwihare, and Azam 2014). Nonetheless, this RDOC proportion is relatively higher than that achieved through short‐term (30 days old) *Synechococcus*‐bacteria coculture (~29%) and axenic *Synechococcus* culture (~26%). Based on the higher proportion of RDOC in the algal‐bacterial coculture at day 30 compared to the axenic culture at day 70, and the increasing RDOC trend in the algal‐bacterial coculture system over time, it is inferred that the RDOC proportion in the coculture system would likely be higher than in the axenic culture at similar time point, such as day 70. This implies that bacterial activity substantially contributes to the conversion of algal‐derived DOC into RDOC. Contrastingly, prior studies have evidenced that DOC released from dead *Synechococcus* cells is highly labile and almost completely degraded by the heterotrophic bacterial community (Wang et al. 2022; Zhao et al. 2019; Zheng et al. 2020). Indeed, the dynamic interaction between living algae and bacteria leads to the release of myriads of known and unknown organic compounds (Liu et al. 2021; Seymour et al. 2017; Uchimiya et al. 2021), a vast number of which may contribute to the marine RDOC pool. For example, interactions between diatom and bacteria and *Phaeocystis* and bacteria are important for the host algal production of carboxyl‐rich alicyclic molecules (CRAM) and humic‐like matter, respectively (Kinsey et al. 2018; Liu et al. 2021). Interestingly, we found that up to 67% of the PPL‐DOC MFs and more than 50% of PPL‐RDOC MFs from the long‐term coexistence between *Syenchococcus* and bacteria shared apparent similarities with in situ surface and deep Pacific Ocean PPL‐DOC MFs. We ensured through meticulous experimental design that the compared MFs clearly originated from *Synechococcus*‐bacteria interactions, without any interference from background natural seawater and exogenous organic carbon molecules. This highlights the significant underestimation of the role of long‐term, persistent algae‐bacteria interactions in shaping the composition and accumulation of the oceanic RDOC reservoir, which plays a pivotal part in global carbon cycling and climate regulation. Although our study focused on *Synechococcus*‐bacteria interactions, the underlying principles of metabolite exchange and carbon dynamics are likely similar across various algae‐bacteria interaction systems. However, further research into other algae‐bacteria systems is necessary for a comprehensive understanding of their RDOC dynamics.

A key revelation from our study is the direct contribution of *Synechococcus* to RDOC genesis, in addition to the well‐established bacterial‐derived RDOC. While previous studies have hinted at the potential release of refractory organic substances by algae including *Synechococcus* (Arakawa et al. 2017; Chrost and Faust 1983; Zhang, Tang et al. 2021; Zhao et al. 2017), we provide the direct experimental evidence for the production and accumulation of diverse RDOC molecules by *Synechococcus* itself. This direct algal RDOC source has previously been obscured by the predominant focus on the bacterial transformation of algae‐derived labile DOC into recalcitrant forms. Intriguingly, the *Synechococcus*‐derived RDOC molecules exhibited consistently higher relative intensity than the bacterial RDOC, suggesting intrinsic differences between the two sources. Mechanistically, we propose two key pathways through which *Synechococcus* can contribute to the RDOC pool: (1) Biosynthetic potential—we show that *Synechococcus* possesses the genetic potential to directly biosynthesize a wide range of potential recalcitrant organic compounds, including mycosporine amino acid conjugates, cyclic lactones, quinolones, and carotenoid derivatives. Previous studies have reported that algal‐derived conjugates of mycosporine amino acids, carotenoids and muramic acids released by marine algae exhibit high recalcitrance and likely contribute to the RDOC pool (Arakawa et al. 2017; Jiao et al. 2014; Raj et al. 2021; Whitehead and Vernet 2000). Moreover, some of these predicted compounds such as hydroxyquinolines have been reported to have strong bactericidal activity, which may further prohibit their degradation (Odingo et al. 2019). Overall, the inability of natural microbial assemblages to degrade these compounds and the identification of genetic potential for the biosynthesis of these compounds by *Synechococcus* confirms the direct release of intrinsically recalcitrant DOC by *Synechococcus*. The consistent accumulation of humic‐like fluorescent components in the *Synechococcus* cultures further corroborates this direct algal RDOC source. (2) Mixotrophic uptake—*Synechococcus* can exhibit mixotrophic growth utilizing simple organic compounds like glucose, and it cannot be ruled out that it may also consume other organic matter (Kang et al. 2004; Ludwig and Bryant 2012). Heterotrophic uptake and release of transformed refractory forms by *Synechococcus* may be an auxiliary pathway analogous to the MCP (Jiao et al. 2014). However, it is unclear whether *Synechococcus* can utilize diverse DOC compounds and, if so, the specific processes and genes mediating uptake and utilization of such DOC in *Synechococcus* require future research. More broadly, many algae are known to synthesize complex metabolites and even exhibit mixotrophic capacity, including bacterial phagocytosis (Pang, Liu, and Liu 2022; Wu et al. 2022). This suggests algae beyond just *Synechococcus* may directly contribute to the ocean RDOC inventories. However, the exact quantitative contribution of direct algal release of RDOC to the total marine RDOC pool remains unknown. Interestingly, we found that nearly half of the RDOC_algae_ MFs identified during the long‐term coexistence of *Synechococcus* and bacteria shared apparent similarities with in situ surface and deep Pacific Ocean PPL‐DOC MFs. As a preliminary calculation, we roughly evaluated the RDOC fluxes from *Synechococcus* alone to be in the range of 0.2–1 Gt C y^−1^ (Data S1) considering its estimated annual carbon fixation rate of 8 Gt C y^−1^, of which 10%–50% is released as DOC and *Synechococcus*‐driven RDOC may account for about one‐fourth of these DOC (Flombaum et al. 2013; Thornton 2014). This is comparable to the annual POC contribution from *Synechococcus* and accounts for 4%–8.3% (Data S1) of the annual ocean carbon sink through the BCP (Boyd et al. 2019; De Martini et al. 2018; Lomas and Moran 2011; Sanders et al. 2014; Stukel et al. 2023). This calculation provides a rough initial estimation, and determining the precise contribution of *Synechococcus* to the oceanic RDOC pool warrants future research.

While *Synechococcus* released unique RDOC molecules, a substantial proportion of the accumulated RDOC was derived from heterotrophic bacteria. The bacterial RDOC pool consisted of structurally conjugated molecules, including steroids, flavonoids, alkaloids, and polyaromatic hydrocarbons, which have been previously associated with marine recalcitrant organic matter fraction (Hertkorn et al. 2013; Jiao et al. 2018; Posada‐Baquero and Ortega‐Calvo 2011). The generation of such diverse, complex compounds from simple algal DOC indicates MCP activities involving extensive structural rearrangement and reworking of organic carbon compounds (Jiao et al. 2010; Lennartz and Dittmar 2022). Additionally, we found that the majority of the potential bacterial RDOC compounds were modified derivatives of DOC molecules including derivatives of benzoic acids, carbohydrates, carboxylic acids, fatty acids, steroids, and others. The presence of these distinct RDOC molecules in long‐term cocultivation is likely due to the structural complexity of the transformed derivatives and their concentration in the surrounding environment, which limits the activity of microbial enzymes and catabolic pathways (Dittmar 2015; Lennartz and Dittmar 2022; Shen and Benner 2022). Low concentrations of individual DOC components have been found to limit their degradation (Arrieta et al. 2015). The molecular complexity of RDOC may make its degradation and utilization thermodynamically unfavorable for some microbial enzyme systems making energy payoff low compared to substrate affinity (Dittmar 2015; Mentges et al. 2019). As the coculture system was viable with *Synechococcus* and the heterotrophic bacterial community continuously growing, there were changes in DOC release albeit at a slow rate, which may enable some heterotrophic members to degrade certain organic compounds until reaching limitations imposed by molecular complexity, concentration, or both. The increased abundance at day 720 of genes involved in complex compound degradation reflects this phenomenon. However, as DOC levels and bacterial abundance increased in our systems, so did the accumulation of specific DOC compounds with recalcitrant signatures. Their persistence despite an actively growing heterotrophic community and their resistance to degradation by natural bacterial assemblages further corroborates the paradigm that the concentration and structural complexity of individual DOC molecules govern their recalcitrance in the ocean (Arrieta et al. 2015).

Moreover, the multivariate factors governing algae‐bacteria‐driven DOC processing and its degradation are challenging to elucidate, yet are imperative for predicting carbon cycling under global change scenarios. Increasing temperature has been shown to favor algae and bacterial metabolism and degradation of some semi‐labile and recalcitrant DOC compounds (Hu et al. 2022; Lønborg et al. 2020). However, the fate of algae‐bacteria‐driven RDOC under the influence of increasing temperature is not yet understood. Here, we provide evidence revealing that ocean warming may accelerate microbial carbon transformations and reduce the accumulation of RDOC through alterations of microbial dynamics and DOC molecular compositions. The elevated temperature exposure stimulated increased productivity and DOC exudation by the *Synechococcus*, evidenced by the increased cell abundance, photosynthetic performance and DOC concentrations. This enhanced supply of fresh, labile DOC in turn likely promoted the metabolism of heterotrophic bacteria to effectively utilize the otherwise non‐bioavailable relatively refractory DOC potentially through the priming effect (Zhang et al. 2023). The decline in the proportions of NOSC and CRAM indices suggests warming promoted transformations of bio‐recalcitrant fractions into more bioavailable forms. This was supported by the higher degradability of the DOC from warming treatments when inoculated with the microbial community. While we did not observe significant changes in microbial community composition at elevated temperatures (Data S1), microbial metabolic function and activity may still be influenced, particularly in their ability to utilize organic compounds. One hypothetical mechanism contributing to these patterns is the “priming effect,” whereby additions of fresh organic matter stimulate microbial mineralization of older relatively recalcitrant carbon (Zhang et al. 2023). Overall, these findings indicate that future ocean warming may negatively affect the ocean RDOC pool by reducing the resident time of potentially refractory DOC molecules thereby jeopardizing the carbon sequestration potential of the marine RDOC pool. Considering the crucial role of recalcitrant DOC reservoirs in global carbon cycling, further research is needed to fully understand the temperature‐driven mechanisms of RDOC turnover and its implications on Earth's climate. It should be noted that while our study revealed significant impacts of future warming on the contribution of RDOC driven by algae‐bacteria interaction, it was conducted over only a seven‐day short period. Future extended studies, especially with long‐term warming treatments, are essential to fully capture the complexities and potential long‐term impact of global warming on the dynamics of RDOC driven by algae‐bacteria interaction.

However, we acknowledge that there are a few limitations to this study. Firstly, the influence of environmental factors like light and pH, which can contribute to DOC transformations were not considered. While light and pH effects are undoubtedly important (Hu et al. 2023), previous studies have demonstrated that microbial processing plays a dominant role in RDOC genesis (Jiao et al. 2010). To address this, we meticulously selected only those RDOC molecules that were common between the long‐term coculture systems and in the dark degradation experiment, suggesting their origin from biotic interaction (Figure 3a). Additionally, light conditions were uniformly maintained (see methods) and pH values were within normal ranges (Data S1). We urge future research to complement the investigation of these environmental parameters. Moreover, the solid phase extraction method may introduce certain biases as it tends to capture more hydrophobic components than hydrophilic ones. Furthermore, dissolved oxygen (DO) saturation and inorganic nutrients (NO_3_^−^, NO_2_^−^ and NH_4_^+^ and PO_4_^3−^) were within normal ranges required for *Synechococcus* growth (Data S1), eliminating their potential inhibitory effects on DOC processing. Analysis of the microbial metagenome suggested the presence of iron siderophore genes, essential for scavenging free iron from the coculture system (Data S1). Future studies could benefit from incorporating metatranscriptomic analyses alongside metagenomic approaches providing a more comprehensive understanding of microbial community functional potential and activity. Additionally, there may be some overlap in molecules released by *Synechococcus* and bacteria, making it harder to differentiate between the RDOC_bact_ and RDOC_algae_. Given the current limitations in identifying RDOC molecular structure, the method used here is a feasible approach to independently identify and compare RDOC_bact_ and RDOC_algae_ molecules. Moreover, conducting long‐term coculture experiments between different algal strains and natural bacterial communities could further expand our conclusions and is worth exploring in future research.

## 8 Conclusion

**FIGURE 7:**
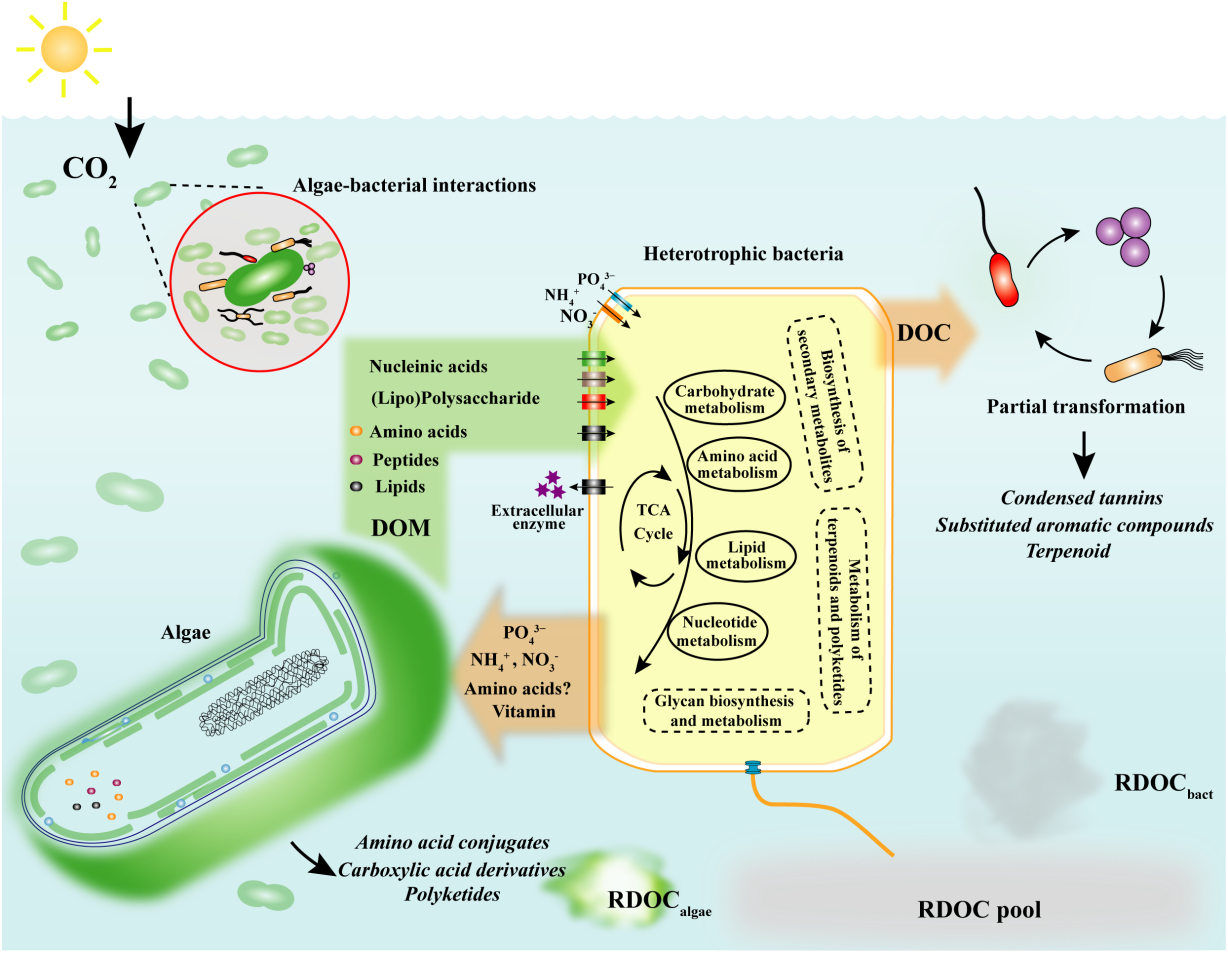
Proposed conceptual model of RDOC accumulation through long‐term algae‐bacteria interactions.

Collectively, our findings reveal that the globally important marine alga *Synechococcus*, in addition to heterotrophic bacteria, can substantially contribute to the genesis and accumulation of the oceanic RDOC pool through both direct release of recalcitrant molecules and long‐term interactions with associated bacteria (Figure 7). The long‐term, continuous interactions between living algae and heterotrophic bacterial communities emerge as a key driver of RDOC dynamics. However, the stability of this crucial carbon reservoir may be threatened by the impacts of climate change. Considering these significant findings, we propose incorporating persistent algae‐bacteria interaction‐driven RDOC production into global carbon cycling models to enable more accurate predictions of carbon budgeting and cycling under global climate change.

## Author Contributions

**Hanshuang Zhao:** conceptualization, data curation, formal analysis, investigation, methodology, validation, visualization, writing – original draft, writing – review and editing. **Zenghu Zhang:** conceptualization, formal analysis, funding acquisition, supervision, validation, visualization, writing – original draft, writing – review and editing. **Shailesh Nair:** conceptualization, data curation, formal analysis, validation, writing – original draft, writing – review and editing. **Hongmei Li:** methodology, validation. **Chen He:** methodology. **Quan Shi:** methodology. **Qiang Zheng:** data curation, writing – review and editing. **Ruanhong Cai:** writing – review and editing. **Genming Luo:** writing – review and editing. **Shucheng Xie:** validation, writing – review and editing. **Nianzhi Jiao:** validation, writing – review and editing. **Yongyu Zhang:** conceptualization, formal analysis, funding acquisition, methodology, project administration, resources, supervision, validation, writing – review and editing.

## Conflicts of Interest

The authors declare no conflicts of interest.

## Supporting information


Figures S1

Tables S1

## Data Availability

The data that support the findings of this study are openly available in figshare at https://doi.org/10.6084/m9.figshare.26057734.v5. The raw 16S rRNA sequences were deposited in the NCBI Sequence Read Archive with the accession number PRJNA982606. The metagenomic raw reads are available in PRJNA978314.
