# Supplementary material for: Overlooked Vital Role of Persistent Algae‐Bacteria Interaction in Ocean Recalcitrant Carbon Sequestration and Its Response to Ocean Warming
Source: Glob Chang Biol. 2024 Nov 27;30(11):e17570. doi: 10.1111/gcb.17570 (PMC11599910; doi:10.1111/gcb.17570)
Supplement: Supplementary file 1 — Figures S1 [file GCB-30-e17570-s002.docx]

**Supplementary Information**

**Overlooked vital role of persistent algae-bacteria interaction in ocean recalcitrant carbon sequestration and its response to ocean warming**

Hanshuang Zhao^1, 2, 3^†, Zenghu Zhang^1, 2, 3^†^*^, Shailesh Nair^1, 2, 3^†, Hongmei Li^1, 2, 3^, Chen He^4^, Quan Shi^4^, Qiang Zheng^5^, Ruanhong Cai^5^, Genming Luo^6^, Shucheng Xie^6^, Nianzhi Jiao^5^, Yongyu Zhang^1, 2, 3*^

^1^Qingdao New Energy Shandong Laboratory, Key Laboratory of Biofuels, Shandong Provincial Key Laboratory of Energy Genetics, Qingdao Institute of Bioenergy and Bioprocess Technology, Chinese Academy of Sciences, Qingdao 266101, China

^2^University of Chinese Academy of Sciences, Beijing 100049, China

^3^Shandong Energy Institute, Qingdao 266101, China

^4^State Key Laboratory of Heavy Oil Processing, China University of Petroleum, Beijing 102249, China

^5^State Key Laboratory of Marine Environmental Science, Xiamen University, Xiamen 361101, China

^6^State Key Laboratory of Biogeology and Environmental Geology, School of Earth Sciences, China University of Geosciences, Wuhan 430074, China

***Correspondence**:

Yongyu Zhang, zhangyy@qibebt.ac.cn; Zenghu Zhang, zhang_zh@qibebt.ac.cn

† Hanshuang Zhao, Zenghu Zhang and Shailesh Nair contributed equally to this work.

**Supplementary Information Guide:**

Supplementary Text

Supplementary Figures S1 to S7

Supplementary Tables S1 to S11

Supplementary Text

Changes in FDOM components during long-term *Synechococcus*-bacteria interaction

Five fluorescent dissolved organic matter (FDOM) components denoted as C1-C5 were identified (Figure S6; Table S5). C1 was categorized as a protein-like component, which is usually considered a labile DOM, while C2, C3, C4 and C5 were classified as humic-like components, which are relatively inert. Both the axenic and coculture systems contained C1, C2 and C4 components. C3 was only present in coculture systems, suggesting its origin from bacterial influence, while C5 was only present in the axenic system. Due to the absence of bacteria, all components a gradual and significant increase (*p_-adj_* <0.05) in the axenic culture systems (Figure S6). Notably, the FDOM components displayed distinct dynamics within the coculture systems (Figure S6). Specifically, component C1 declined rapidly due to bacterial utilization, whereas component C2 declined initially for 180 days before gradually increasing until the end of the experiment. In contrast, components C3 and C4 increased consistently, showing resistance to bacterial utilization. These suggested that C1 may represent labile LDOC and C2 may represent semi-recalcitrant DOC. Meanwhile, the gradual increase in C3 and C4 is likely indicative of RDOC, potentially derived from the bacteria or algae bacteria interaction and algae, respectively.


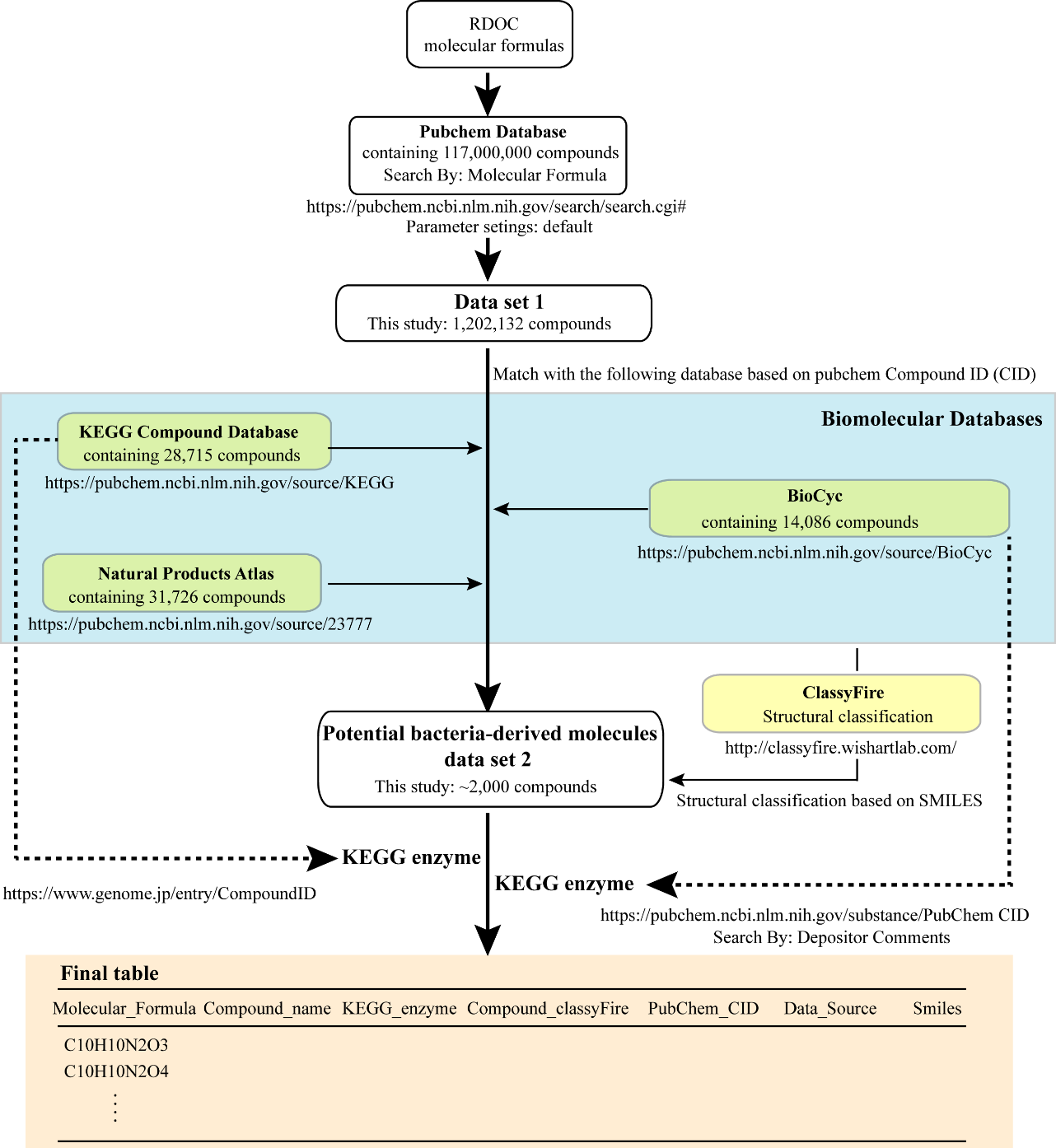


**Figure S1. A flowchart depicting the steps involved in predicting the structure of RDOC molecules and identifying their potentially related genes.** The RDOC molecular formulas (MFs) were searched against the PubChem chemical database (https://pubchem.ncbi.nlm.nih.gov/search/search.cgi) using default settings. The resultant compound structures were then constructed into a dataset and screened against the KEGG compound (https://www.genome.jp/kegg/), BioCyc (https://www.biocyc.org/), and the Natural Products Atlas (https://www.npatlas.org/) databases to remove matches with synthetic origin. The remaining potential natural compound structures were then taxonomically classified using ClassyFire (http://classyfire.wishartlab.com) with ChemOnt v.2.1 databases. Finally, the above predicted compound names and related information, as well as the enzyme commission (EC) numbers of compounds obtained from KEGG and BioCyc, were manually organized into a table.


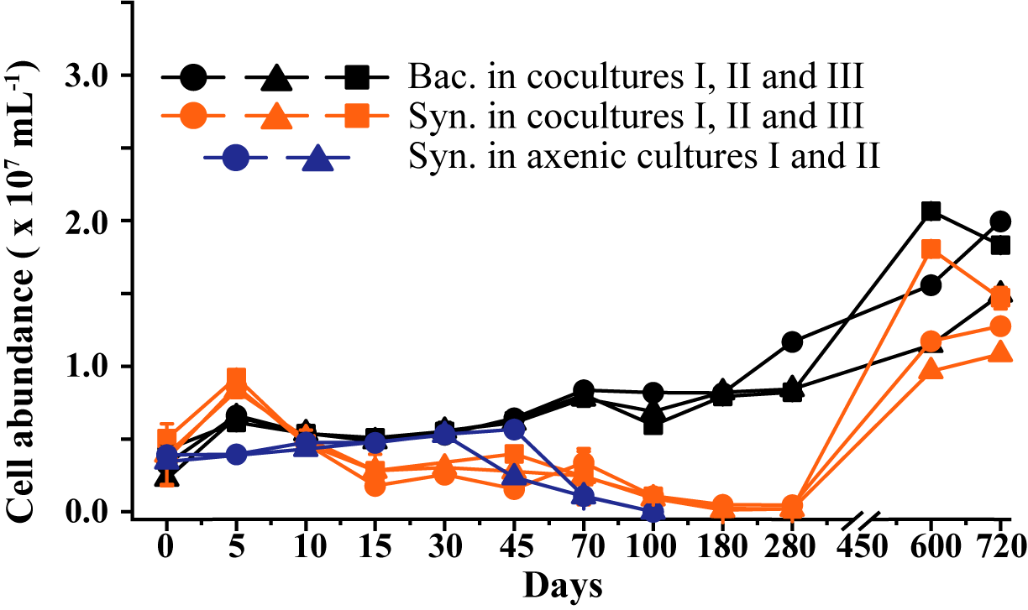


**Figure S2. Mean cell abundance of *Synechococcus* and heterotrophic bacteria over time in cocultures and axenic cultures.** Error bars indicate standard deviation (SD, *n* = 3). The black line represents bacterial abundance while the orange line represents the *Synechoccoccus* abundance in the coculture systems. The blue line represents *Synechococcus* abundance in axenic culture systems.


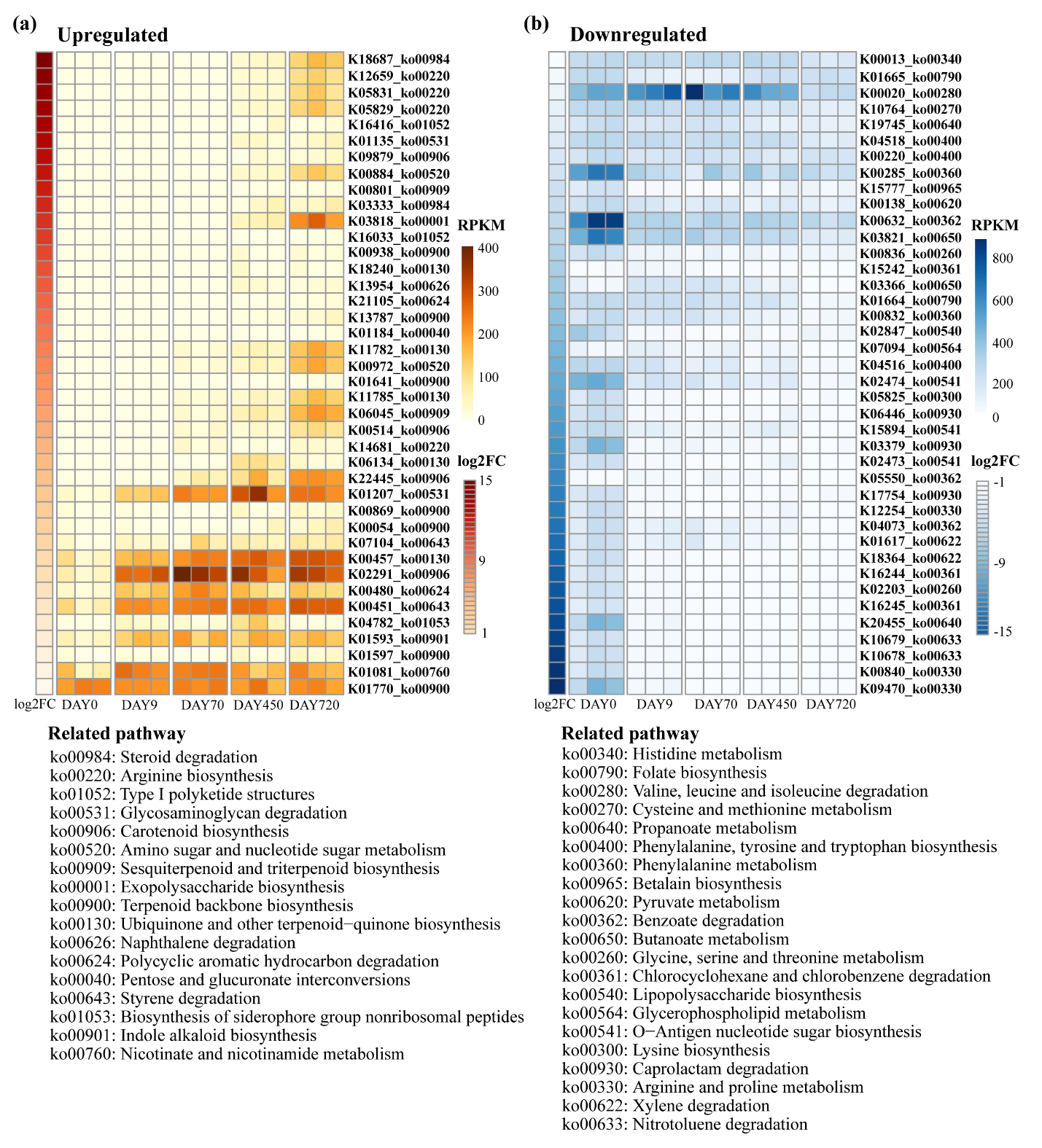


**Figure S3. Abundance of differentially expressed functional genes in long-term *Synechococcus*-bacteria cocultures.** Heatmaps showing **(a)** upregulated and **(b)** downregulated bacterial genes (KEGG orthology, KOs) and their associated pathways in cocultures at different time points (DAY0, DAY9, DAY70, DAY450, and DAY720). All functional genes displayed in the heatmap were significantly (*p* < 0.05) differentially abundant according to DESeq2 analysis. Color intensity indicates gene abundance measured in reads per kilobase per million mapped reads (RPKM). Each time point includes *n* = 3 biological replicates.


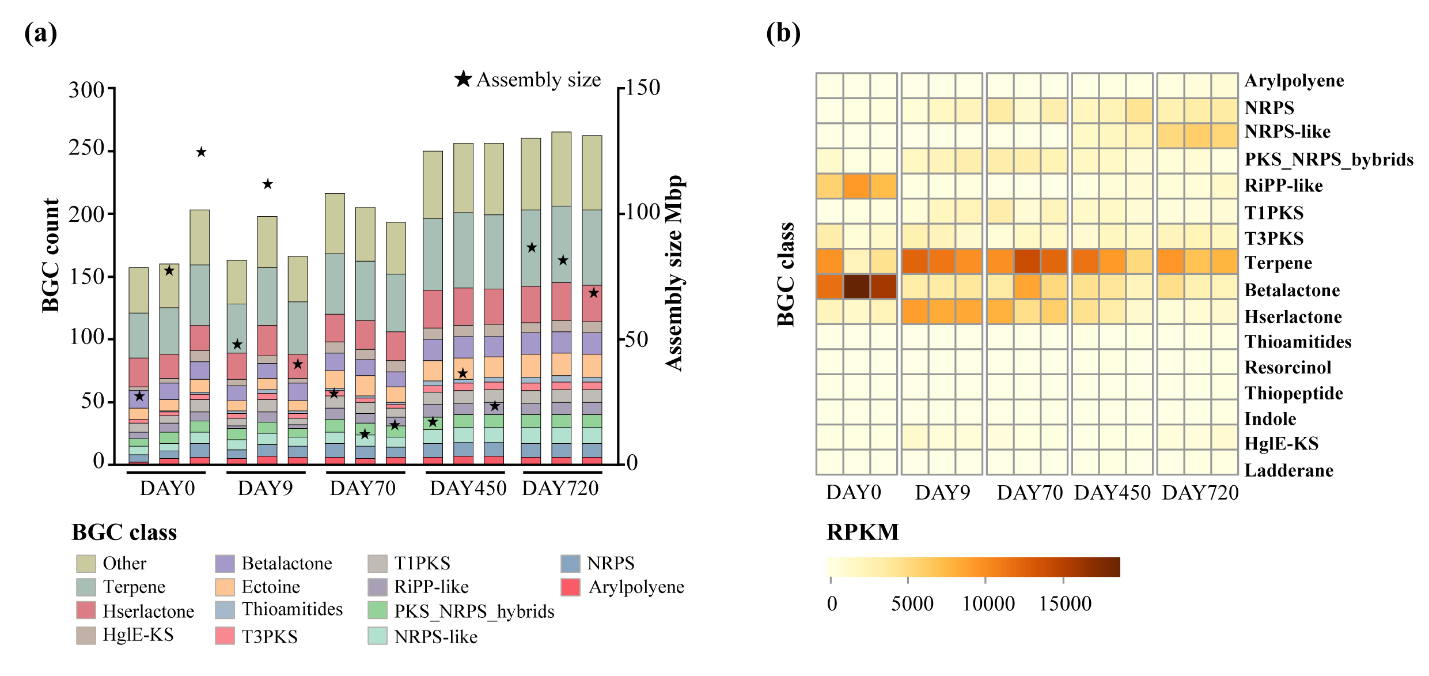


**Figure S4. Diversity and abundance of biosynthetic gene clusters (BGCs) in *Synechococcus*-bacteria coculture over time. (a)** The number of BGC classes in coculture at different time points (DAY0, DAY9, DAY70, DAY450, and DAY720). The color scale represents BGC classes; the asterisk denotes the assembly size of total BGC corresponding to each sample in Mb. **(b)** Heatmap displaying the RPKM-normalized abundance of BGCs in cocultures over time. Each row represents a BGC class and each column corresponds to a different time point (DAY0, DAY9, DAY70, DAY450, and DAY720). Each time point includes *n* = 3 biological replicates.


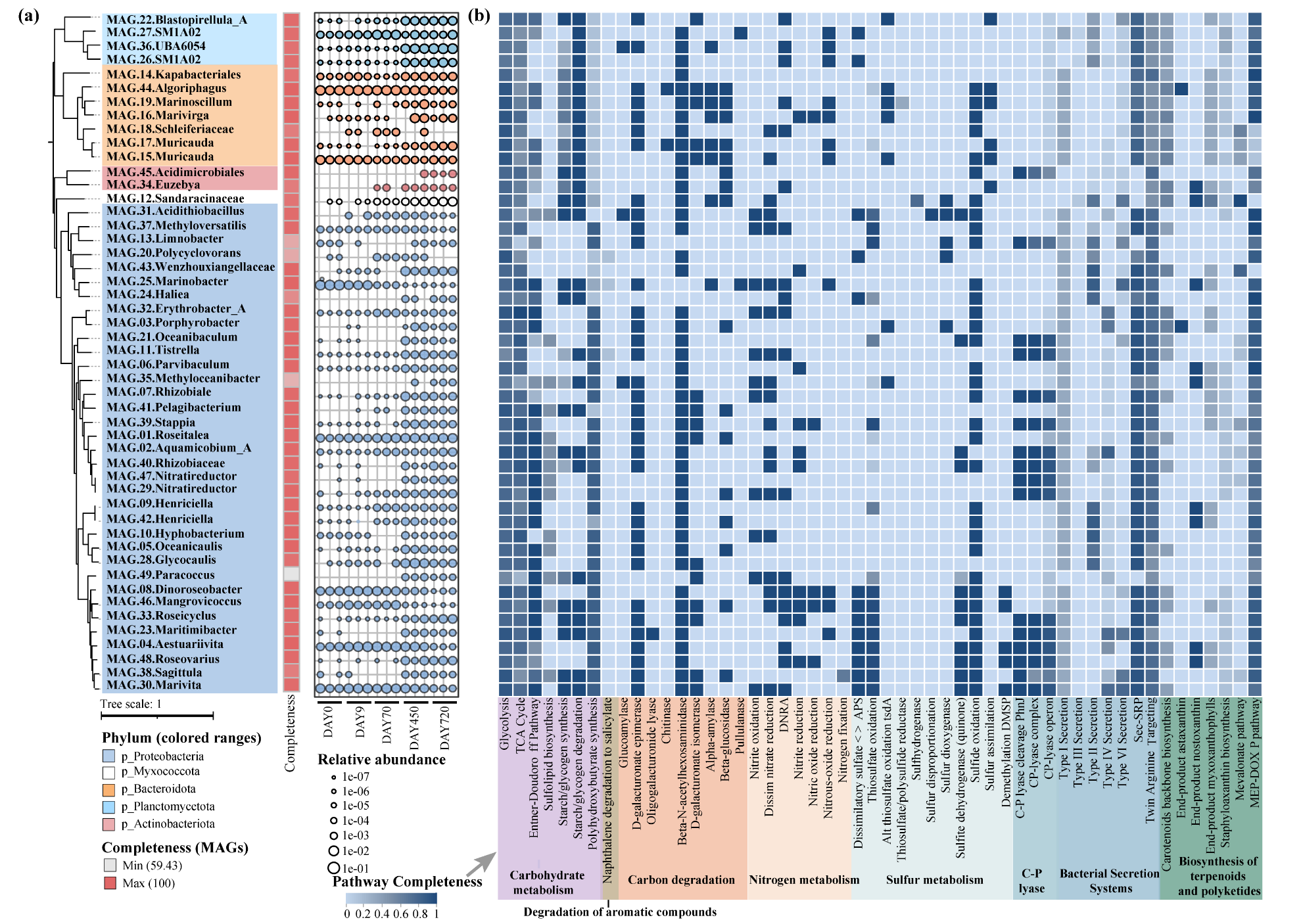


**Figure S5. Genomic and functional annotation of 49 metagenome‑assembled genomes (MAGs)**. (**a**) The phylogenetic tree was constructed using IQ-TREE based on 120 concatenate marker genes from the MAGs. The taxonomic classification of each MAG is shown, with tree branches colored according to phylum. The corresponding boxes on the right of the phylogenetic tree represent the percent completeness of each MAG. The following bubble plot represents the relative abundance of each corresponding MAG across different culture time points (DAY0, DAY9, DAY70, DAY450, and DAY720). Bubble size is proportional to the relative abundance of each MAG and bubble color indicates the corresponding MAG phylum. (**b**) Heatmap showing the completeness of metabolic pathways in each MAG as determined by KEGG Decoder. Each row corresponds to the MAG described in the phylogenetic tree and each column represents a metabolic pathway, ordered by metabolic class. The intensity of the blue color indicates the percent completeness of each pathway based on the presence or absence of key genes. Dark blue represents a complete or nearly complete pathway, while light blue represents an absent or incomplete pathway.


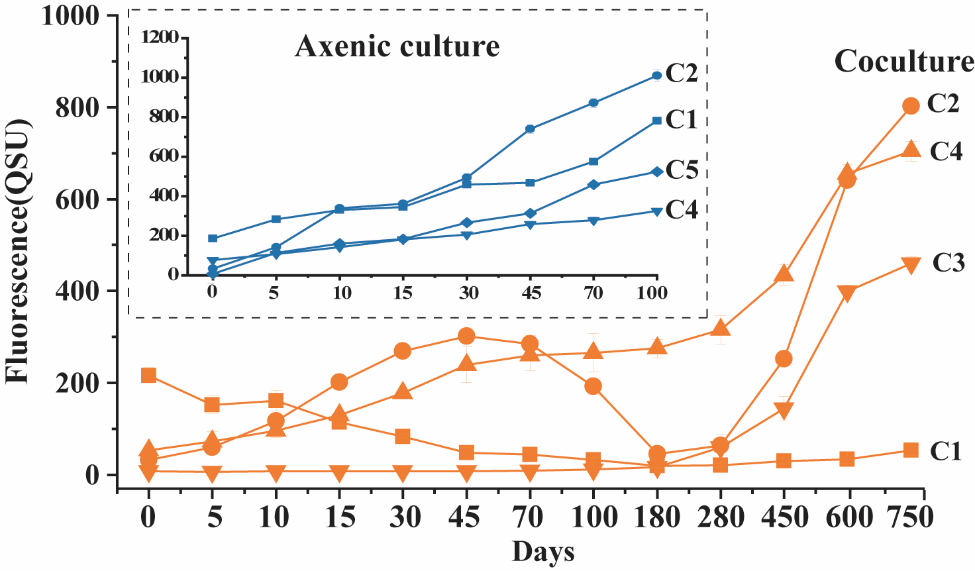


**Figure S6. FDOM intensity over time in axenic and coculture systems measured by excitation-emission matrix parallel factor analysis (EEM-PARAFAC).** Component C1 represents protein-like FDOM and components C2, C3, C4, and C5 represent humic-like FDOM. The coculture system is represented by an orange line, while the axenic culture system is represented by a blue line. Error bars indicate SD (*n* = 3).


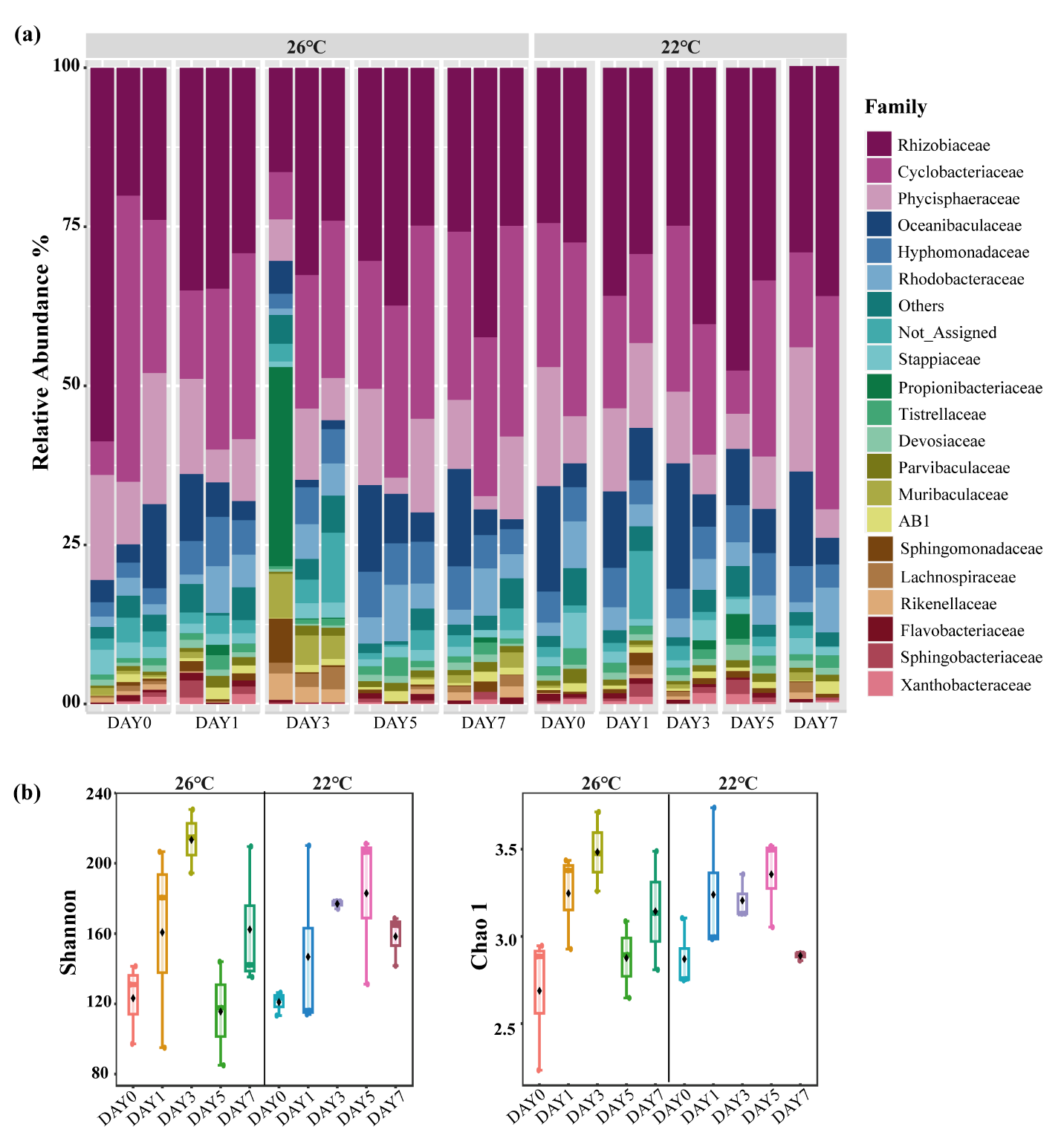


**Figure S7. Taxonomic compositions and alpha diversity of bacterial communities in the ocean warming conditions.** (**a**) Stacked barplot showing the relative abundances of bacterial communities in warming (26℃) groups and control (22℃) groups at the family level. The warming groups include triplicate samples (*n* = 3), and the control groups include duplicate samples (*n* = 2). (**b**) Boxplot showing the alpha diversity indices, including Shannon and Chao 1, for different treatments.

**Supplementary Tables:**

**Table S1.** FT-ICR MS identified DOC molecular characteristics from the long-term algae-bacteria coculture, axenic culture systems and microbial degradation experiments.

**Table S2.** Summary of the molecular characteristics of *Synechococcus* and bacteria produced RDOC molecules.

**Table S3.** Summary compounds corresponding to RDOC_bact_ molecular formulas predicted using KEGG, BioCyc and Natural Products Atlas databases.

**Table S4.** Abundance of differentially expressed bacterial genes and related pathways in long-term *Synechococcus*-bacteria coculture systems. Color intensity indicates gene abundance measured in reads per kilobase per million mapped reads (RPKM). Brown = upregulated genes, Green = downregulated genes. Rep1-3: replicate samples.

**Table S5.** Characteristics of identified FDOM components in the long-term algae-bacteria coculture and axenic culture.

**Table S6.** Summary of compounds corresponding to RDOC_algae_ molecular formula predicted using public databases.

**Table S7.** *Synechococcus* PCC7002 functional genes potentially involved in the synthesis of RDOC compounds and their gene abundance measured in reads per kilobase per million mapped reads (RPKM). Rep1-3: replicate samples.

**Table S8.** FT-ICR MS identified DOC molecular characteristics from the surface and deep seawater of the western Pacific Ocean.

**Table S9.** Estimated RDOC contribution of *Synechococcus* to oceanic RDOC pool.

**Table S10.** Inorganic nutrient concentrations, DO percentage and pH values in the long-term cocultur experiments. Means and standard deviation derived from triplicate samples.

**Table S11.** Functional genes involved in iron metabolism in the long-term algae-bacteria coculture experiments and their gene abundance measured in reads per kilobase per million mapped reads (RPKM). Rep1-3: replicate samples.
